# Peripartal treatment with low‐dose sertraline accelerates mammary gland involution and has minimal effects on maternal and offspring bone

**DOI:** 10.14814/phy2.15204

**Published:** 2022-03-02

**Authors:** Celeste M. Sheftel, Luma C. Sartori, Emily R. Hunt, Robbie S. J. Manuel, Autumn M. Bell, Rafael R. Domingues, Lella A. Wake, Brandon R. Scharpf, Chad M. Vezina, Julia F. Charles, Laura L. Hernandez

**Affiliations:** ^1^ Molecular and Cellular Pharmacology Training Program University of Wisconsin‐Madison Madison Wisconsin USA; ^2^ Department of Animal and Dairy Sciences University of Wisconsin‐Madison Madison Wisconsin USA; ^3^ Department of Orthopedic Surgery Brigham and Women’s Hospital, and Harvard Medical School Boston Massachusetts USA; ^4^ Department of Comparative Biosciences University of Wisconsin‐Madison Madison Wisconsin USA; ^5^ Department of Medicine Brigham and Women’s Hospital, and Harvard Medical School Boston Massachusetts USA

**Keywords:** bone, lactation, mammary gland, serotonin, SSRI

## Abstract

Women mobilize up to 10% of their bone mass during lactation to provide milk calcium. About 8%–13% of mothers use selective serotonin reuptake inhibitors (SSRI) to treat peripartum depression, but SSRIs independently decrease bone mass. Previously, peripartal use of the SSRI fluoxetine reduced maternal bone mass sustained post‐weaning and reduced offspring bone length. To determine whether these effects were fluoxetine‐specific or consistent across SSRI compounds, we examined maternal and offspring bone health using the most prescribed SSRI, sertraline. C57BL/6 mice were given 10 mg/kg/day sertraline, from the beginning of pregnancy through the end of lactation. Simultaneously, we treated nulliparous females on the same days as the primiparous groups, resulting in age‐matched nulliparous groups. Dams were euthanized at lactation day 10 (peak lactation, *n* = 7 vehicle; *n* = 9 sertraline), lactation day 21 (weaning, *n* = 9 vehicle; *n* = 9 sertraline), or 3m post‐weaning (*n* = 10 vehicle; *n* = 10 sertraline) for analysis. Offspring were euthanized at peak lactation or weaning for analysis. We determined that peripartum sertraline treatment decreased maternal circulating calcium concentrations across the treatment period, which was also seen in nulliparous treated females. Sertraline reduced the bone formation marker, procollagen 1 intact N‐terminal propeptide, and tended to reduce maternal BV/TV at 3m post‐weaning but did not impact maternal or offspring bone health otherwise. Similarly, sertraline did not reduce nulliparous female bone mass. However, sertraline reduced immunofluorescence staining of the tight junction protein, zona occludens in the mammary gland, and altered alveoli morphology, suggesting sertraline may accelerate mammary gland involution. These findings indicate that peripartum sertraline treatment may be a safer SSRI for maternal and offspring bone rather than fluoxetine.

## Graphical abstract

Provides images of the mammary gland. There is also immunofluorescence staining.

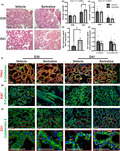

## 1 INTRODUCTION

Mineralization of the infant skeleton during development requires substantial calcium intake, from milk (Wysolmerski, 2010). To stimulate maternal calcium resorption from trabecular bones to supply the milk calcium, the mammary gland secretes parathyroid hormone‐related protein (PTHrP) into circulation. Circulating PTHrP stimulates osteoclastic bone resorption and osteolytic osteolysis, increasing serum calcium for mammary gland uptake, and subsequent secretion into milk (Liu et al., 2012; Miyamoto et al., 2019; VanHouten et al., 2003). Increased PTHrP production by the mammary gland is stimulated by serotonin (5HT; 5‐hydroxytryptamine), and 5HT has been demonstrated to increase gene and protein expression of calcium transporters in the mammary gland, supporting a role for 5HT signaling in the regulation of maternal calcium homeostasis (Hernandez et al., 2012; Laporta, Keil, Vezina, et al., 2014; Sheftel & Hernandez, 2020).

Women who exclusively breastfeed one baby lose an average of 210 mg of calcium per day, resulting in up to 10% reduced bone mineral density (BMD) over the course of the recommended 6 months of breastfeeding (Kovacs, 2016; Ryan & Kovacs, 2019). Maternal BMD was previously believed to return to pre‐lactation levels 12 months after weaning (Kovacs, 2016; Ritchie et al., 1998; Sowers et al., 1993). However, recent epidemiological studies have challenged this paradigm. One study determined that a reduction in bone density persisted ≥2.6 years after the cessation of lactation (Bjornerem et al., 2017), and other studies have associated the cumulative amount of time breastfeeding with postmenopausal bone deficits (Hwang et al., 2016; Kim et al., 2015; Mgodi et al., 2015; Okyay et al., 2013; Rojano‐Mejia et al., 2011).

Antidepressants, such as selective serotonin reuptake inhibitors (SSRIs), are commonly used during pregnancy and lactation, both by women already being diagnosed with clinical depression prior to becoming pregnant and those who developed depression during and after pregnancy (Gavin et al., 2005; Marcus et al., 2003). SSRIs are considered the first‐choice treatment for prenatal and postpartum depression (Andrade et al., 2008; Cooper et al., 2007; Davanzo et al., 2011). Although the primary role of SSRIs is inhibiting 5HT reuptake transporter SERT in the brain, thereby increasing neuronal 5HT exposure and regulating mood and behavior, 95% of body 5HT is synthesized in the periphery. SSRIs can therefore enhance peripheral 5HT signaling by inhibiting peripheral SERT, as well as increasing 5HT synthesis and decreasing 5HT degradation (Marshall et al., 2014).

Nonneuronal 5HT regulates diverse physiological processes, including platelet aggregation, glucose metabolism, bone homeostasis, and immune function (Martin et al., 2017; Rapport et al., 1948; Walther et al., 2003; Yadav et al., 2008). SSRIs have been shown to decrease BMD independent of lactation in the general population (Bonnet et al., 2007; Ortuno et al., 2016; Tsapakis et al., 2012). Previously, we demonstrated that peripartum fluoxetine treatment results in a sustained reduction of maternal BMD compared to pregnancy and lactation alone (Weaver et al., 2018). Additionally, in utero and lactational exposure to fluoxetine reduced offspring long bone length and reduced offspring head circumference (Weaver et al., 2019). Together, this raises concerns about long‐term bone health among women who are prescribed an SSRI during pregnancy and lactation and how exposure to an SSRI impacts the developing offspring.

Different SSRIs, and different pharmacological doses of SSRIs, have been shown to have differential effects on bone microarchitecture in non‐lactation models and have disparate effects on the developing offspring when taken throughout pregnancy (Koren & Nordeng, 2012; Kumar et al., 2018). Given our previous study with fluoxetine demonstrating reduced maternal bone mass as well as reduced offspring bone formation (Weaver et al., 2018, 2019), it raised the question whether these effects are fluoxetine‐specific or consistent across the class of SSRIs. Therefore, we investigated the most prescribed peripartum SSRI, sertraline (Zoloft^™^), on maternal calcium trafficking and BMD to determine if maternal bone mass effects vary among SSRIs. We hypothesized that sertraline, like fluoxetine, would result in an increase in mammary calcium trafficking and a subsequent sustained reduction of maternal BMD. Understanding how different SSRIs impact the maternal skeleton during lactation can have therapeutic implications for treatment of peripartum depression, potentially resulting in one SSRI being prescribed over another due to the potential reduced negative impact on bone.

## 2 METHODS

### 2.1 Animals

**FIGURE 1:**
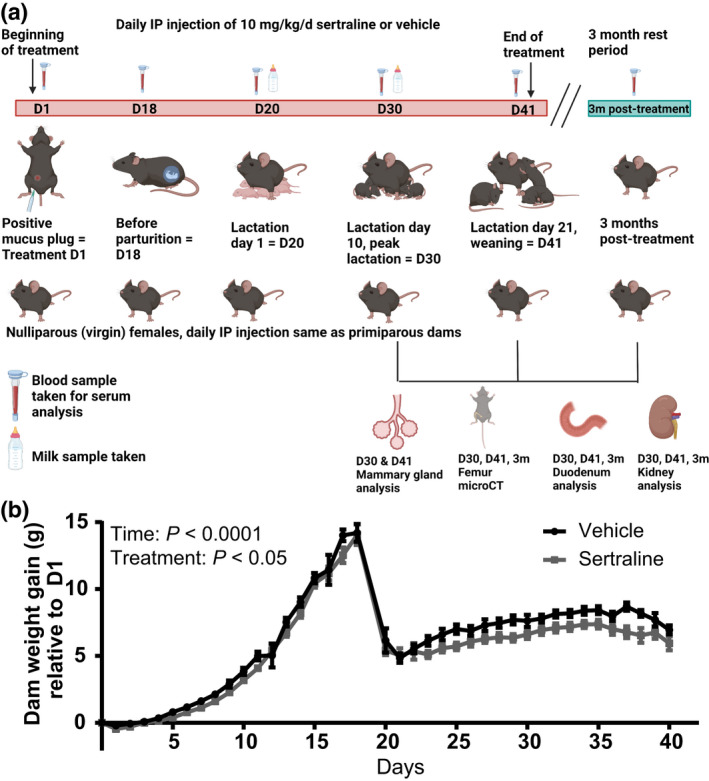
Peripartum sertraline reduces maternal weight gain throughout lactation. Treatment scheme depicted in (a). C57BL/6 dams and nulliparous virgin females were administered vehicle (8.3% DMSO) or sertraline (10 mg/kg/day in 8.3% DMSO) from the day of conception or treatment day 1 (D1) through end of lactation (D41) and then rested for 3 months post‐weaning (3m post‐treatment). Blood was collected from all mice, 6 h after treatment, on D1, D18, D20, D30, and D41 and after a 6 h fast on 3 months post‐treatment (post‐weaning). Milk samples were taken at the start of lactation, D20, or peak lactation, D30. Dams were euthanized at D30, D41, or 3m post‐treatment and the mammary gland, femur, duodenum, and kidney were harvested. Dam weight (g) was measured, weight gain was measured by correcting the dam's daily weight to the weight at D1 (b). Dams were harvested at peak lactation (D30, treatment day 30) (*n* = 7 vehicle; *n* = 9 sertraline), weaning (D41, treatment day 41) (*n* = 9 vehicle; *n* = 9 sertraline), or were aged an additional 3 months after weaning (3m post‐treatment) (*n* = 10 vehicle; *n* = 10 sertraline). Data presented as mean ± SEM and analyzed using two‐way ANOVA for treatment and time

All experiments were performed under protocol number A005789‐01 and approved by the Research Animal Care and Use Committee at the University of Wisconsin‐Madison. Female C57BL/6 mice from our mating colony were housed in a controlled environmental facility for biological research in the Animal and Dairy Sciences Department vivarium at the University of Wisconsin‐Madison at a temperature of 25°C, 50%–60% humidity, and a 12‐h light:12‐h dark cycle, with ad libitum food and water. Beginning at 6 weeks of age, female mice were bred overnight, and observation of a seminal plug signified successful copulation and beginning of pregnancy, at which time the mice were housed individually and randomly assigned to sertraline treatment or vehicle control. Beginning at the date of observation of seminal plug (D1), the dams received daily intraperitoneal injections of either 10 mg/kg bodyweight sertraline hydrochloride (Sigma‐Aldrich, #S6319) reconstituted in 8.3% DMSO in saline or sterile vehicle. This dose causes calvarial bone deficits in mice (Howie et al., 2018). The final volume injected into each dam was 0.12 ml of either sertraline or vehicle. Injections were performed daily between 0800 and 0900 h from plug day (D1) through weaning (D41). Nulliparous virgin females of the same age were treated on the same dates as the primiparous females for a total of 41 days. Dams and pups were weighed daily at the time of injection. Dams were either harvested at peak lactation (D30, treatment day 30) (*n* = 7 vehicle; *n* = 9 sertraline), weaning (D41, treatment day 41) (*n* = 9 vehicle; *n* = 9 sertraline), or were aged an additional 3 months after weaning (3m post‐treatment) (*n* = 10 vehicle; *n* = 10 sertraline). The dams collected at D30 were ~10 ± 2 weeks of age (2.5–3 months), at D41 were ~12 ± 2 weeks of age (3 months), and the mice aged to 3 months post‐termination of treatment were ~25 ± 2 weeks of age (6 months). Pups born to primiparous dams were harvested at D30 (postnatal day 10) or at D41 (postnatal day 21, weaning) of treatment. Nulliparous virgin mice were used as a control and harvested at treatment day 30 (*n* = 9 vehicle; *n* = 9 sertraline), treatment day 41 (*n* = 10 vehicle; *n* = 10 sertraline), or 3 months post‐treatment (*n* = 10 vehicle; *n* = 10 sertraline). Treatment scheme denoted in Figure 1a.

### 2.2 Sample collection

Milk consumption per pup was determined daily throughout lactation using the weigh‐suckle‐weigh (WSW) method (Laporta et al., 2013). Milk was collected on lactation day 1 (treatment D20) and peak lactation (D30). Milk letdown was initiated with 300 µL of subcutaneous oxytocin injection, then mice were anesthetized by isoflurane inhalation, followed by manual expression of milk. Milk was frozen at −80°C until analysis. Litters were not standardized, due to a reduced litter size in sertraline‐treated dams. The litter size and pup mortality data for this study were recently published (Domingues et al., 2022).

Mice were fasted for 6 h prior to blood collection, which occurred on baseline (D1), before parturition (D18), lactation day 1 (D20), peak lactation (D30), and weaning (D41). Blood was collected from the submandibular vein using a 5.5‐mm lancet, then was placed on ice for 20 min to allow disruption of platelets, centrifuged at 1500 *g* at 4°C for 20 min to isolate serum, which was stored at −80°C until analysis.

At peak lactation (D30), weaning (D41), or 3 months post‐treatment the dams were fasted for 6 h and then euthanized via carbon dioxide inhalation followed by cervical dislocation. The number 4 mammary gland was collected, fixed overnight in 10% formalin, and stored in 70% ethanol until paraffin embedding for histological examination. The duodenum, kidneys, one femur, and all other mammary glands were rapidly extracted and immediately snap frozen in liquid nitrogen to preserve tissue integrity and stored at −80°C until analysis. The carcass was collected for microCT evaluation by storing in 70% ethanol.

### 2.3 MicroCT analysis

Dam and nulliparous femurs were analyzed by microCT using a Scanco Medical μCT 35 system with an isotropic voxel size of 7 μm. Scans were conducted in 70% ethanol using an x‐ray tube potential of 55 kVp, an x‐ray intensity of 0.145 mA and an integration time of 400 ms. Femoral length was measured on scout scans using digital calipers. A region beginning 0.14 mm proximal to the growth plate and extending 1.4 mm proximally was selected for cancellous bone analysis. A second region 0.6 mm in length and centered at the midpoint of the femur was used to calculate cortical parameters. A semi‐automated contouring approach was used to distinguish cortical and trabecular bone. The region of interest was threshold using a global threshold that set the bone/marrow cutoff at 512 mgHA/cm^3^ for trabecular bone and 871.8 mgHA/cm^3^ for cortical bone. Three‐dimensional microstructural properties of bone, including bone volume fraction (BV/TV), trabecular thickness (Tb.Th), trabecular number (Tb.N.), trabecular separation (Tb.Sp.), midshaft bone volume fraction (M.BV/TV), and cortical thickness (C.Th) were calculated using software supplied by the manufacturer reported according to consensus guidelines on rodent microCT (Bouxsein et al., 2010).

Offspring femur microCT analysis used the same equipment and settings as above. Femoral length was measured on scout scans using digital calipers. A region beginning just proximal to the growth plate and extending 1.05 mm proximally was selected for cancellous bone analysis. A second region 0.42 mm in length and centered at the midpoint of the femur was used to calculate cortical parameters. A semi‐automated contouring approach was used to distinguish cortical and trabecular bone. The region of interest was threshold using a global threshold that set the bone/marrow cutoff at 385.8 mgHA/cm^3^ for trabecular bone and 491.5 mgHA/cm^3^ for cortical bone. Three‐dimensional microstructural properties of bone were calculated as above.

### 2.4 Assays

5HT concentrations were determined using a 5HT Enzyme Immunoassay Kit (Beckman Coulter, #IM1749) using serum diluted 1:150 or 150 µg protein in radioimmunoprecipitation lysis buffer as previously described (Laporta, Keil, Vezina, et al., 2014; Weaver et al., 2018). Serum procollagen I intact N‐terminal (PINP) concentrations were determined using the PINP Enzyme Immunoassay Kit (Immunodiagnostic Systems, #AC‐33F1) using serum diluted 1:10 according to the manufacturer's instructions. Serum collagen type 1 cross‐linked C‐telopeptide (CTX‐I) concentrations were determined using a RatLaps CTX Enzyme Immunoassay Kit (Immunodiagnostics Systems, #AC‐06F1) using serum undiluted according to the manufacturer's instructions. Total calcium was determined using a Calcium Assay Kit (Cayman Chemical Company, #701220) in serum diluted 1:2 or in tissue using 100 µg protein in 1% triton lysis buffer according to the manufacturer's instructions. Mammary gland intracellular cAMP concentrations were determined using a cyclic AMP XP Assay Kit (Cell Signaling Technology, #4339S) using 50 µg total protein in 1% triton lysis buffer according to the manufacturer's instructions. Sertraline concentrations were determined using a Sertraline Enzyme Immunoassay Kit (Neogen Toxicology, #131319) using serum diluted 1:5 according to the manufacturer's instructions. All assays had an intra‐assay CV of <10% and inter‐assay CV of <5%.

### 2.5 Mammary gland, duodenum, kidney and femur protein extraction, RNA, and RT‐qPCR

Protein was extracted from the mammary gland, kidney, and duodenum using radioimmunoprecipitation assay buffer (1X PBS, 1% octyl phenoxypolyethoxylethanol [NP‐40], 0.5% sodium deoxycholate, 0.1% SDS) or 1% triton lysis buffer supplemented, with 10 µl/ml Halt Protease and Phosphatase Inhibitor Cocktail (Thermo Fisher Scientific, #78441). Lysates were homogenized and cleared by centrifugation for 15 min at 12,000 *g*. Protein concentration was determined using bicinchoninic acid assay (Bioworld, #20831001‐1).

RNA was extracted from the mammary gland, kidney, duodenum, and entire femurs using TRI‐Reagent (Molecular Research, Thermo Fisher Scientific, #NC9277980) according to manufacturer's protocol. RNA of 1 µg was reverse transcribed with High‐Capacity cDNA Reverse Transcription Kit (Applied Biosystems, Thermo Fisher Scientific, #4368814) with murine RNase inhibitor (New England Biolabs, #M0314L). Quantitative RT‐PCR was conducted using the CFX96 Touch‐Real‐Time PCR Detection System (Bio‐Rad Laboratories). Reaction mixtures and cycling conditions were performed as previously described (Laporta et al., 2013). Primers were designed with an optimal annealing temperature of 60°C. Amplification efficiencies of primers were accepted within a range of 95%–105% and a singular melt‐curve. The primer sequences are listed in Table 1. The housekeeping parameter was the geometric mean of *Rsp9* and *S15*, with mammary glands using *Rsp9*, *S15*, and *K8* (to control for mammary epithelial cells). Analysis was conducted using the 2^−∆∆CT^ method. See Table S1 for primer sequences.

**TABLE 1:** Primiparous femoral trabecular bone parameters evaluated by MicroCT

	Measurements						*p*‐value		
	D30		D41		3m				
	VEH	SRT	VEH	SRT	VEH	SRT	Time	Treatment	Interaction
Tb. connective density	216.4 ± 15	240.3 ± 9.6	182.1 ± 35.5	137.4 ± 8.9	56.2 ± 11.3	30.7 ± 2.5	<0.0001	0.22	0.093
Tb.N (1/mm)	4.2 ± 0.07	4.4 ± 0.06	4.2 ± 0.3	3.7 ± 0.1	2.6 ± 0.3	2.3 ± 0.07	<0.0001	0.16	0.21
Tb.Sp. (mm)	0.24 ± 0.004	0.23 ± 0.003	0.24 ± 0.02	0.27 ± 0.007	0.39 ± 0.04	0.42 ± 0.01	<0.0001	0.19	0.37
Tb.Th (mm)	0.033 ± 0.0004	0.034 ± 0.0004	0.038 ± 0.002	0.039 ± 0.002	0.042 ± 0.002	0.041 ± 0.002	<0.0001	0.77	0.72
Tissue BMD (mg Hg/cm^3^)	68.3 ± 6.3	76.1 ± 3.05	80.0 ± 10.3	57.7 ± 5.0	28.2 ± 7.5	14.4 ± 4.3	<0.0001	0.078	0.058
Tissue TMD (mg Hg/cm^3^)	998.1 ± 4.1	994.9 ± 3.3	1000.0 ± 4.8	1004.9 ± 3.3	1035.8 ± 5.1	1047.7 ± 8.9	<0.0001	0.3	0.36

Note

Dams were harvested at peak lactation (D30, treatment day 30) (*n* = 6 vehicle; *n* = 7 sertraline), weaning (D41, treatment day 41) (*n* = 6 vehicle; *n* = 6 sertraline), or were aged an additional 3 months after weaning (3m post‐treatment) (*n* = 5 vehicle; *n* = 6 sertraline). Data presented as mean ± SEM and analyzed using two‐way ANOVA for treatment and time. *p* < 0.05 is considered significant and 0.1 < *p* < 0.05 is considered a tendency.

Abbreviations: BMD, bone mineral density; SRT, sertraline; Tb.N., trabecular number; Tb.Sp., trabecular spacing; Tb.Th., trabecular thickness; TMD, total mineral density; VEH, vehicle.

### 2.6 Mammary gland histology and immunofluorescence

Sectioned mammary glands were stained with hematoxylin and eosin (H&E), with 1 section per animal. 20× images were captured using a Zeiss Axio Vert. A1 microscope and Q‐capture pro 7 software. Three nonoverlapping images per animal were then used to quantify alveoli number and size using ImageJ software. Mammary gland slides were deparaffinized and processed for immunofluorescence using the following antibodies: TPH1 (Abcam, #ab228588, 1:250) or Ki67 (Abcam, #ab15580, 1:200) in combination with the epithelial cell marker E‐Cadherin (BD Biosciences, #610182, 1:250) and ZO‐1 (Invitrogen, #33‐9100, 1:50) in combination with the epithelial cell marker cytokeratin 8 (Abcam, #ab53280, 1:100). Secondary antibodies were incubated for 1 h at room temperature and diluted 1:250 using Alexa Fluor 594 Goat Anti‐Rabbit IgG (Life Technologies, #A11037), Alexa Fluor 488 Goat Anti‐Mouse IgG (Life Technologies, #A11001), Alexa Fluor 594 Goat Anti‐Mouse IgG (Life Technologies, #A11005), and Alexa Fluor 488 Goat Anti‐Rabbit IgG (Jackson ImmunoResearch, #111‐545–144). Nuclei were visualized using DAPI, dilactate (4’,6‐diamidino‐2‐phenylindole, dilactate) (Invitrogen, #D3571, 300 nM final concentration). All images were captured using an Eclipse E600 compound microscope (Nikon Instruments Inc.) fitted with a 20× dry objective (Plan Fluor NA = 0.75; Nikon) and equipped with a CoolSNAP Dyno CCD camera (Teledyne Photometrics), and NIS elements imaging software (Nikon Instruments Inc.).

### 2.7 Statistics

All statistical analyses were conducted using GraphPad Prism 9 (Version 9.2.0). Analysis between two treatments without the effect of time were performed using a Student's unpaired two‐sided *t*‐test. When data were not normally distributed, a Mann–Whitney test was performed for nonparametric data. Analyses with multiple *t*‐tests, FDR was used to correct for multiple comparisons. Analyses with multiple time points were conducted using a two‐way ANOVA with Tukey's multiple comparisons test to detect differences between treatment groups. For all analyses, differences among means were considered significant if *p* < 0.05 or a tendency if 0.05 < *p* < 0.1. All values are reported as mean ± SEM.

## 3 RESULTS

### 3.1 Maternal circulating, but not mammary or duodenal, 5HT is impacted by sertraline treatment

**FIGURE 2:**
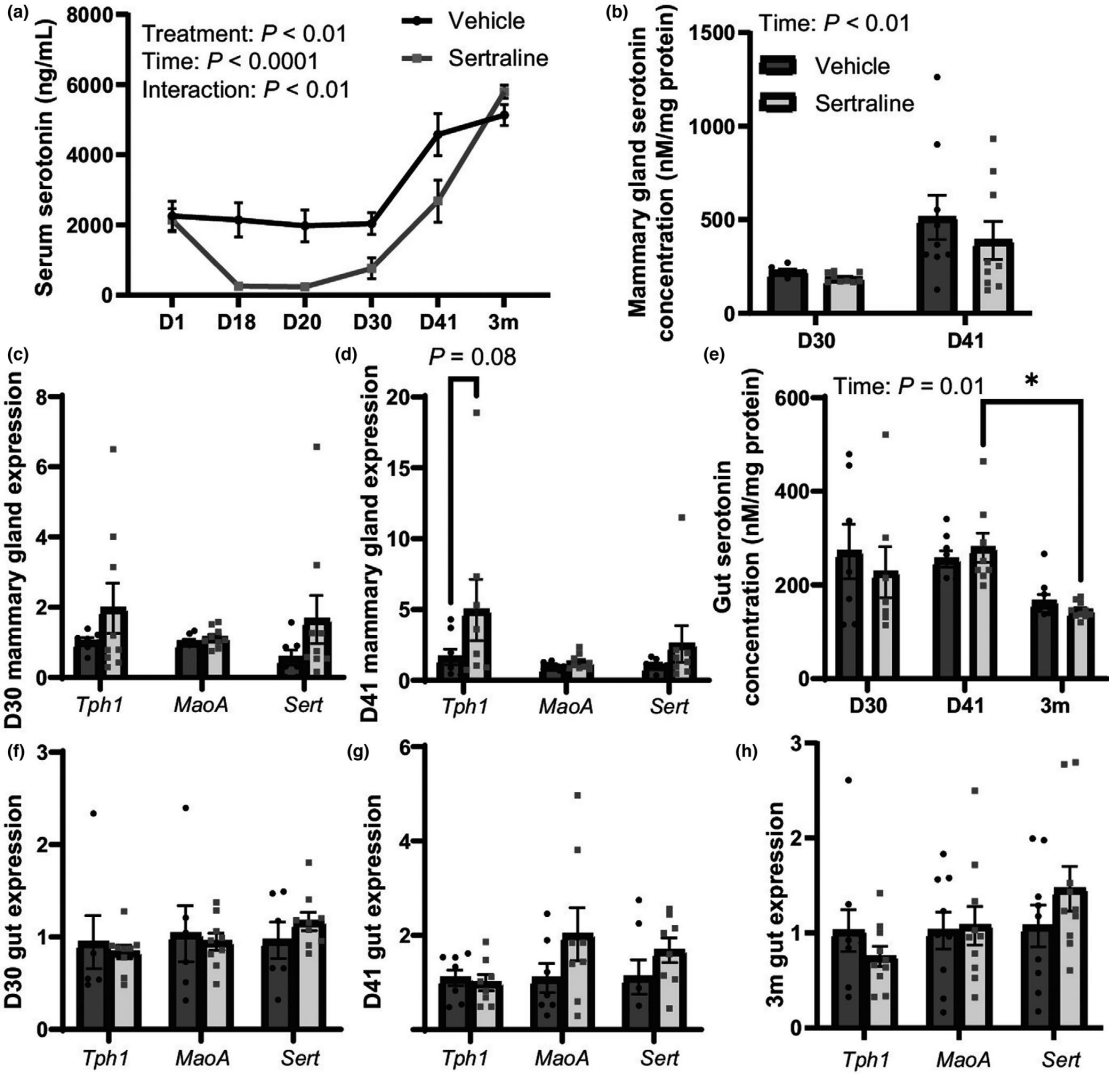
Peripartum sertraline decreased circulating serotonin but did not impact tissue serotonin. C57BL/6 dams were administered vehicle (8.3% DMSO) or sertraline (10 mg/kg/day in 8.3% DMSO) from the day of conception, treatment day 1 (D1) through end of lactation (D41) and then rested for 3 months post‐weaning (3m post‐treatment). Blood was collected from all mice, 6 h after treatment, on D1, D18, D20, D30, and D41 and at D30, D41, or 3m dams were euthanized, and serum was isolated. Serum serotonin concentrations were measured at each timepoint (a). Mammary gland serotonin concentration (mM/mg protein) was measured at D30 and D41, during lactation (b) and genes involved in serotonin metabolism were measured at D30 (c) and D41 (d). Duodenum serotonin concentration (mM/mg protein) was measured at each timepoint (e), along with genes involved in serotonin metabolism at D30 (f), D41 (g), and 3m post‐treatment (h). Dams were harvested at peak lactation (D30, treatment day 30) (*n* = 7 vehicle; *n* = 9 sertraline), weaning (D41, treatment day 41) (*n* = 9 vehicle; *n* = 9 sertraline), or were aged an additional 3 months after weaning (3m post‐treatment) (*n* = 10 vehicle; *n* = 10 sertraline). Data presented as mean ± SEM and analyzed using two‐way ANOVA for treatment and time or *t*‐test with multiple comparisons. *p < 0.05

C57BL/6 dams were treated daily with 10 mg/kg/day sertraline beginning at confirmation of pregnancy (pregnancy day 1, treatment D1) throughout end of lactation where pups reached weaning at 21 days of lactation (treatment D41) (treatment schematic Figure 1a). Maternal weight was lower in sertraline‐treated dams, particularly throughout lactation (*p* < 0.05, Figure 1b). We next examined how peripartum sertraline impacted maternal 5HT homeostasis. Maternal circulating 5HT concentration was decreased by sertraline treatment throughout pregnancy and lactation (*p* < 0.01, Figure 2a). These findings are consistent with previous studies showing decreased circulating 5HT with SSRI treatment (Bismuth‐Evenzal et al., 2012; Holck et al., 2019; Rossum et al., 2020).

We then measured 5HT concentration and 5HT synthesis genes in tissues known to secrete 5HT into circulation, the mammary gland, and intestine. Mammary tissue 5HT concentration was unchanged by sertraline treatment when compared to vehicle on D30 and D41 (Figure 2b), but there was a time effect, with 5HT concentrations significantly increased at weaning (D41) compared to peak lactation (D30) (*p* < 0.01). *Tph1* (the rate‐limiting enzyme in 5HT synthesis) gene expression was twofold higher in the treatment dams compared to controls on D30 (Figure 2c), increasing by fivefold in the on D41 (*p* = 0.08, Figure 2d).

Duodenal 5HT concentration was unchanged by sertraline treatment (Figure 2e). However, 5HT concentrations were significantly altered over time (*p* < 0.01) with a reduction observed in sertraline‐treated dams by 3m post‐treatment (*p* < 0.05). We did not observe changes in expression of 5HT metabolism target genes *Tph1*, *MaoA*, or *Sert* at any timepoint (Figure 2f–h).

### 3.2 Maternal sertraline treatment impacts maternal calcium metabolism

**FIGURE 3:**
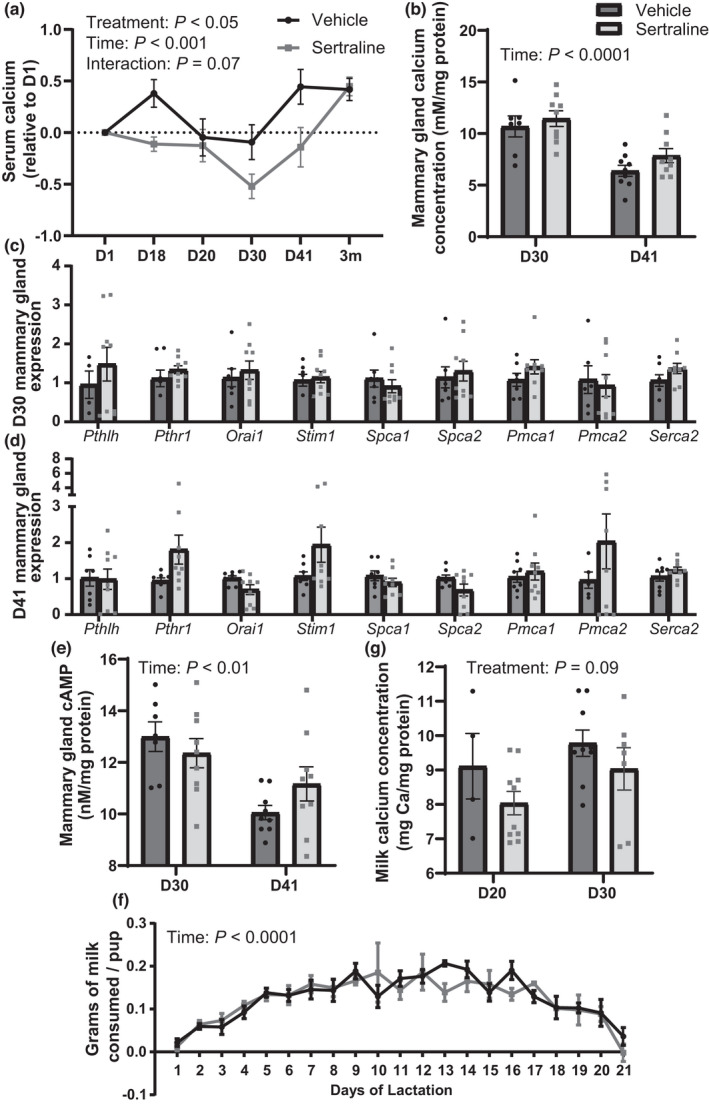
Peripartum sertraline reduced circulating calcium and tended to reduce milk calcium without impacting pup milk consumption. Serum samples taken 6 h after treatment, were used to measure circulating calcium (mM), which is shown corrected to each mouse's baseline (D1) sample (a). Mammary gland calcium concentration (mM/mg protein) was measured at D30 and D41, during lactation (b) and genes involved in calcium trafficking were measured at D30 (c) and D41 (d). Mammary cAMP, a measure of PTHrP activity was measured during lactation (e). Each day of lactation, pups were removed from the dam and fasted for 4 h, after the fast, the pups were weighed, returned to the dam for a 1‐h nursing bout, and then weighed again. The difference in weight represented the amount of milk consumed for each litter, which was then divided by the total number of pups in the litter on that given day, to estimate the grams of milk consumed per pup (f). Milk calcium was determined (mg Ca) corrected to the milk protein (mg protein) at the first day of lactation, D20, and peak lactation, D30 (g). Dams were harvested at peak lactation (D30, treatment day 30) (*n* = 7 vehicle; *n* = 9 sertraline), weaning (D41, treatment day 41) (*n* = 9 vehicle; *n* = 9 sertraline), or were aged an additional 3 months after weaning (3m post‐treatment) (*n* = 10 vehicle; *n* = 10 sertraline). Data presented as mean ± SEM and analyzed using two‐way ANOVA for treatment and time or *t*‐test with multiple comparisons

Based on previous findings by our group demonstrating that 5HT increases calcium transport into the mammary gland, we tested if sertraline treatment increases calcium transport into the mammary gland. Circulating calcium relative to D1 was significantly decreased by sertraline treatment (*p* < 0.05) and changed over time (*p* < 0.001, Figure 3a). This finding contrasts with our previous fluoxetine study where we observed increased maternal circulating calcium with SSRI treatment (Weaver et al., 2018).

We then analyzed the mammary gland and milk to determine whether decreased circulating calcium could impact transfer of calcium into milk and therefore the offspring. Mammary gland calcium content was unchanged by sertraline treatment compared to vehicle (Figure 3b), but there was an overall significant reduction in mammary gland calcium content on D41 compared to D30 (*p* < 0.0001). Mammary *Pthlh* (PTHrP gene) and its receptor, *Pthr1* were unchanged by sertraline on D30 (Figure 3c), along with the calcium transporters (*Orai1*, *Stim1*, *Spca1*, *Spca2*, *Pmca1*, *Pmca2*, and *Serca2*), suggesting sertraline treatment does not impact calcium trafficking at peak lactation. Similarly, calcium trafficking genes were not impacted by sertraline on D41 (Figure 3d). Mammary cAMP concentrations, a common measure of PTHrP activity (Figure 3e), was not significantly altered by sertraline treatment, but cAMP concentrations were reduced overall on D41 compared to D30 (*p* < 0.01).

We found no differences in milk production, as measured by WSW, between treatment and control (Figure 3f), but there was a significant change over time in both vehicle‐ and sertraline‐treated dams achieving peak lactation around D30 with a steady decline after peak lactation (*p* < 0.0001). Milk calcium (Figure 3g) tended to be decreased by sertraline treatment (mg calcium/mg protein) (*p* = 0.09), but there was no time effect and no treatment by time interaction (*p* > 0.05).

### 3.3 Sertraline treatment may result in faster involution of the mammary gland

**FIGURE 4:**
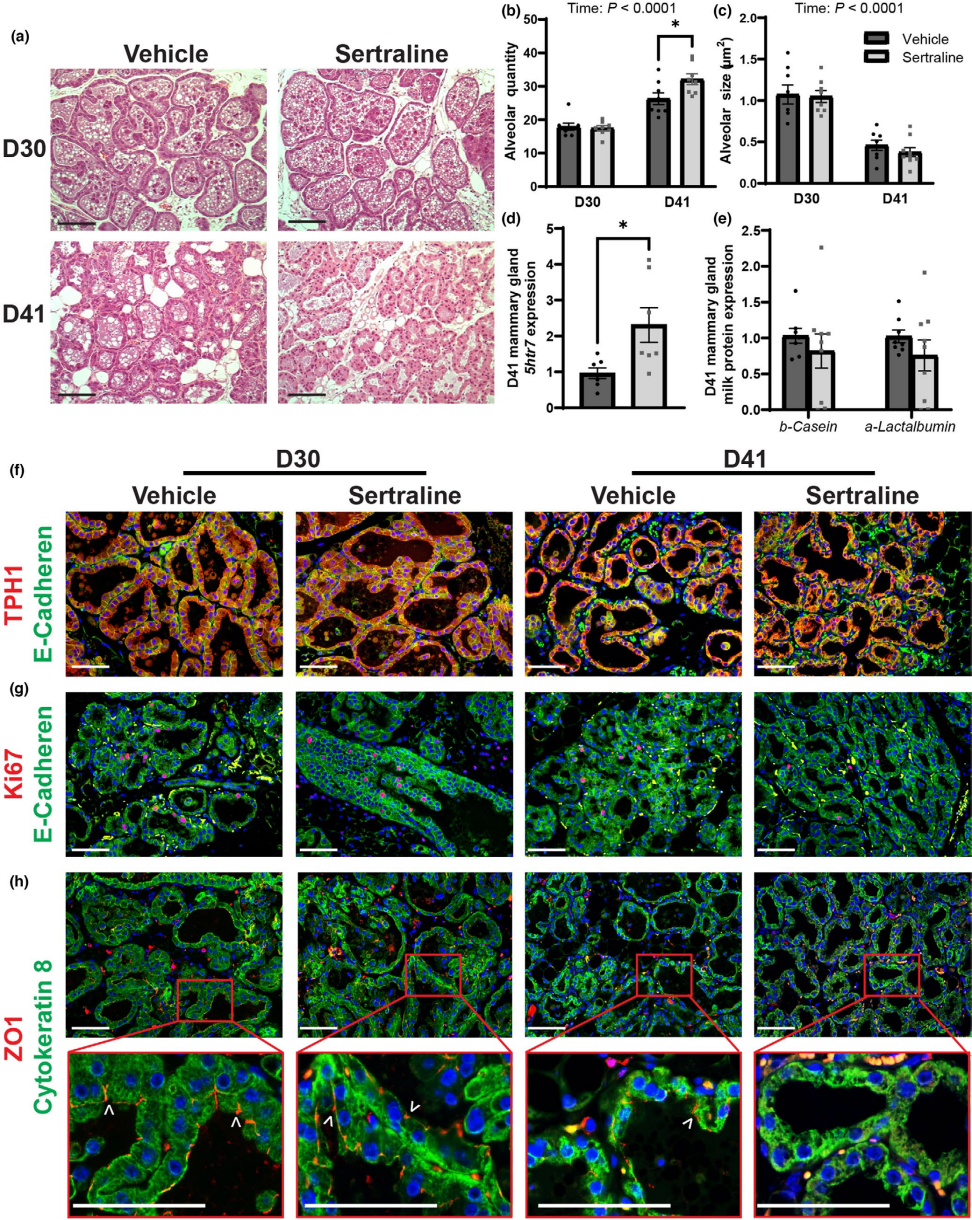
Peripartum sertraline does not impact the mammary gland at peak lactation but may increase involution at weaning. The right inguinal mammary gland was collected, fixed in 10% formalin, and used for histological examination. Histological hematoxylin and eosin stained mammary glands at D30 and D41 (a). The number of alveoli (b) and the diameter of the alveoli (c) were quantified from three nonoverlapping sections for each mouse. Mammary gland expression of *5htr7* (d) and milk protein genes *α*‐*lactalbumin* and *β*‐*casein* (e) were examined at weaning. The number 4 mammary gland was also used for immunofluorescence, imaged at 20× magnification at D30 and D41. Tph1 (red) combined with E‐cadherin (green) and dapi (blue) (f), Ki67 proliferation marker (red) combined with E‐cadherin (green) and dapi (blue) (g), and ZO1 tight junction protein (red) combined with cytokeratin 8 (green) and dapi (blue), white arrows denoting positive ZO1 staining (h). Mammary gland sections were imaged at 20× magnification, scale bars represent 100 µm. Dams were harvested at peak lactation (D30, treatment day 30) (*n* = 7 vehicle; *n* = 9 sertraline), weaning (D41, treatment day 41) (*n* = 9 vehicle; *n* = 9 sertraline), or were aged an additional 3 months after weaning (3m post‐treatment) (*n* = 10 vehicle; *n* = 10 sertraline). Data presented as mean ± SEM and analyzed using two‐way ANOVA for treatment and time or *t*‐test with multiple comparisons. *p < 0.05

We measured alveolar size, area of the alveoli lumen, and quantity, number of alveoli in each picture, by hematoxylin and eosin staining of mammary glands (Figure 4a–c). No differences in alveolar quantity were observed between the vehicle and sertraline on D30. We also investigated the possibility of accelerated mammary gland involution due to SSRI exposure (Marshall et al., 2010), which normally occurs as offspring begin to rely less on milk for growth and more on dry food. We examined the differences in mammary gland alveoli morphology between peak lactation (D30) and weaning (D41), as involution is associated with decreased alveoli diameter and increased re‐differentiation of adipocytes (Marshall et al., 2010). On D41, the number of alveoli increased (*p* < 0.05) with sertraline treatment. Between D30 and D41, we observed a time effect, resulting in a significant increase in alveolar number (*p* < 0.001) and a decrease in alveolar diameter (*p* < 0.0001) by D41. We measured expression of the 5HT receptor seven gene (*5htr7*) as it has been shown to regulate mammary gland involution (Marshall et al., 2010; Pai et al., 2015; Pai & Horseman, 2008). A twofold increase in *5htr7* expression (Figure 4d) with sertraline treatment (*p* < 0.05) was observed. However, this was not accompanied by alterations in milk protein gene expression of *β*‐*casein* or *ɑ*‐*lactalbumin* (Figure 4e). We therefore examined the localization of the tight junction protein, zona occludens 1 (ZO‐1), the proliferation marker, Ki67, and the rate‐limiting enzyme in 5HT synthesis, TPH1 at D30 and D41 to determine whether sertraline accelerated involution (Figure 4f–h). TPH1 staining was present in both treatments at both timepoints, as expected, since 5HT is necessary for both lactation and involution. Ki67 staining was reduced by D41 in both treatments, which was further reduced by sertraline treatment. We then examined ZO‐1, a tight junction scaffolding protein that becomes disrupted during involution. ZO‐1 staining decreased by D41 in both vehicle and sertraline dams, suggesting both were undergoing involution, but ZO‐1 staining in sertraline‐ treated dams was undetectable, suggesting sertraline‐treated dams may have increased tight junction permeability compared to vehicle‐treated dams on D41 of treatment.

### 3.4 Maternal sertraline treatment does not exacerbate lactation‐induced bone loss

**FIGURE 5:**
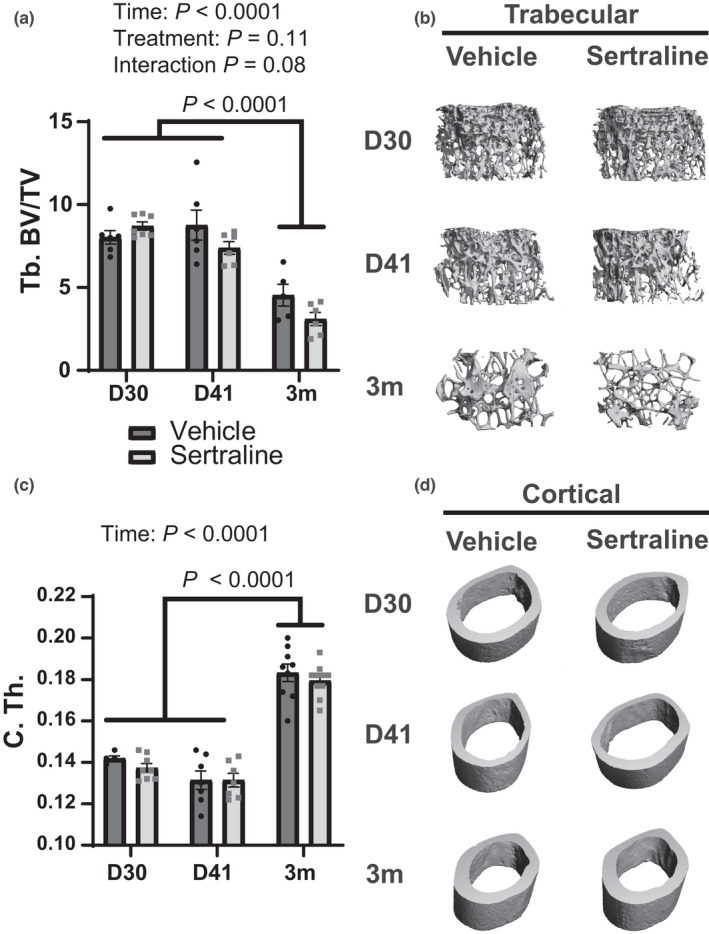
Peripartum sertraline does not impact maternal trabecular or cortical bone. Trabecular (Tb.) bone volume/total volume (BV/TV) was examined at D30, D41, or 3m post‐treatment (a) and a three‐dimensional reconstruction image of the trabecular bone are shown (b). Cortical (C.) thickness was measured (c) and the three‐dimensional reconstruction image of the cortical bone are shown (d). Dams were harvested at peak lactation (D30, treatment day 30) (*n* = 7 vehicle; *n* = 9 sertraline), weaning (D41, treatment day 41) (*n* = 9 vehicle; *n* = 9 sertraline), or were aged an additional 3 months after weaning (3m post‐treatment) (*n* = 10 vehicle; *n* = 10 sertraline). Data presented as mean ± SEM and analyzed using two‐way ANOVA for treatment and time

Previously, fluoxetine resulted in a sustained reduction of maternal bone mass (Weaver et al., 2018). Lactation is associated with a 20%–30% decrease in maternal BMD in rodents (VanHouten & Wysolmerski, 2003), so failing to regenerate bone mass afterwards is a significant detriment to skeletal integrity. To examine whether sertraline had similar effects, we examined bone structural parameters by microCT and assessed bone metabolism by determining relative expression of a panel of key bone metabolic genes. MicroCT analysis was used to examine the effect of sertraline treatment on maternal bone mass throughout lactation and post‐weaning. Femoral trabecular bone volume / total volume (BV/TV) was unchanged by sertraline treatment but was significantly reduced by 3m post‐treatment in both vehicle and sertraline dams (*p* < 0.0001, Figure 5a). Femoral trabecular BV/TV was not impacted by sertraline treatment (*p* > 0.05) but there was a tendency for a reduction at 3m post‐weaning (*p* = 0.08). There was a tendency for reduced femoral trabecular BMD by sertraline treatment (*p* = 0.08), but no other sertraline effects on femoral trabecular bone (Table 1). Similarly, there were no effects of sertraline treatment on cortical bone (Table 2), but cortical thickness increased by 3m post‐treatment in both vehicle and sertraline dams (*p* < 0.001, Figure 5b). By 3m post‐treatment all dams exhibited age‐related skeletal changes with decreased number and increased spacing of trabecular bone (Table 1), but cortical porosity was only reduced in vehicle dams (Table 2).

**TABLE 2:** Primiparous femoral cortical bone parameters evaluated by MicroCT

	Measurements						*p*‐value		
	D30		D41		3m				
	VEH	SRT	VEH	SRT	VEH	SRT	Time	Treatment	Interaction
Periosteal area (mm^2^)	1.87 ± 0.02	1.84 ± 0.05	1.82 ± 0.03	1.75 ± 0.03	1.84 ± 0.02	1.86 ± 0.03	0.092	0.4	0.41
Periosteal Porosity (mm)	9.2 ± 0.06	9.04 ± 0.2	8.9 ± 0.08	8.7 ± 0.1	8.6 ± 0.06	8.7 ± 0.08	0.0006	0.47	0.34
Tissue BMD (mg Hg/cm^3^)	442.9 ± 5.9	423.8 ± 7.4	416.8 ± 15.8	419.4 ± 9.4	577.04 ± 15.8	565.4 ± 4.9	<0.0001	0.32	0.65
Tissue TMD (mg Hg/cm^3^)	1204.2 ± 7.4	1189.8 ± 5.1	1216 ± 7.3	1214.9 ± 6.0	1281.7 ± 12.0	1281.5 ± 5.9	<0.0001	0.36	0.48

Note

Dams were harvested at peak lactation (D30, treatment day 30) (*n* = 5 vehicle; *n* = 8 sertraline), weaning (D41, treatment day 41) (*n* = 7 vehicle; *n* = 7 sertraline), or were aged an additional 3 months after weaning (3m post‐treatment) (*n* = 9 vehicle; *n* = 10 sertraline). Data presented as mean ± SEM and analyzed using two‐way ANOVA for treatment and time. *p* < 0.05 is considered significant and 0.1 < *p* < 0.05 is considered a tendency.

Abbreviations: BMD, bone mineral density; SRT, sertraline; TMD, total mineral density; VEH, vehicle.

**FIGURE 6:**
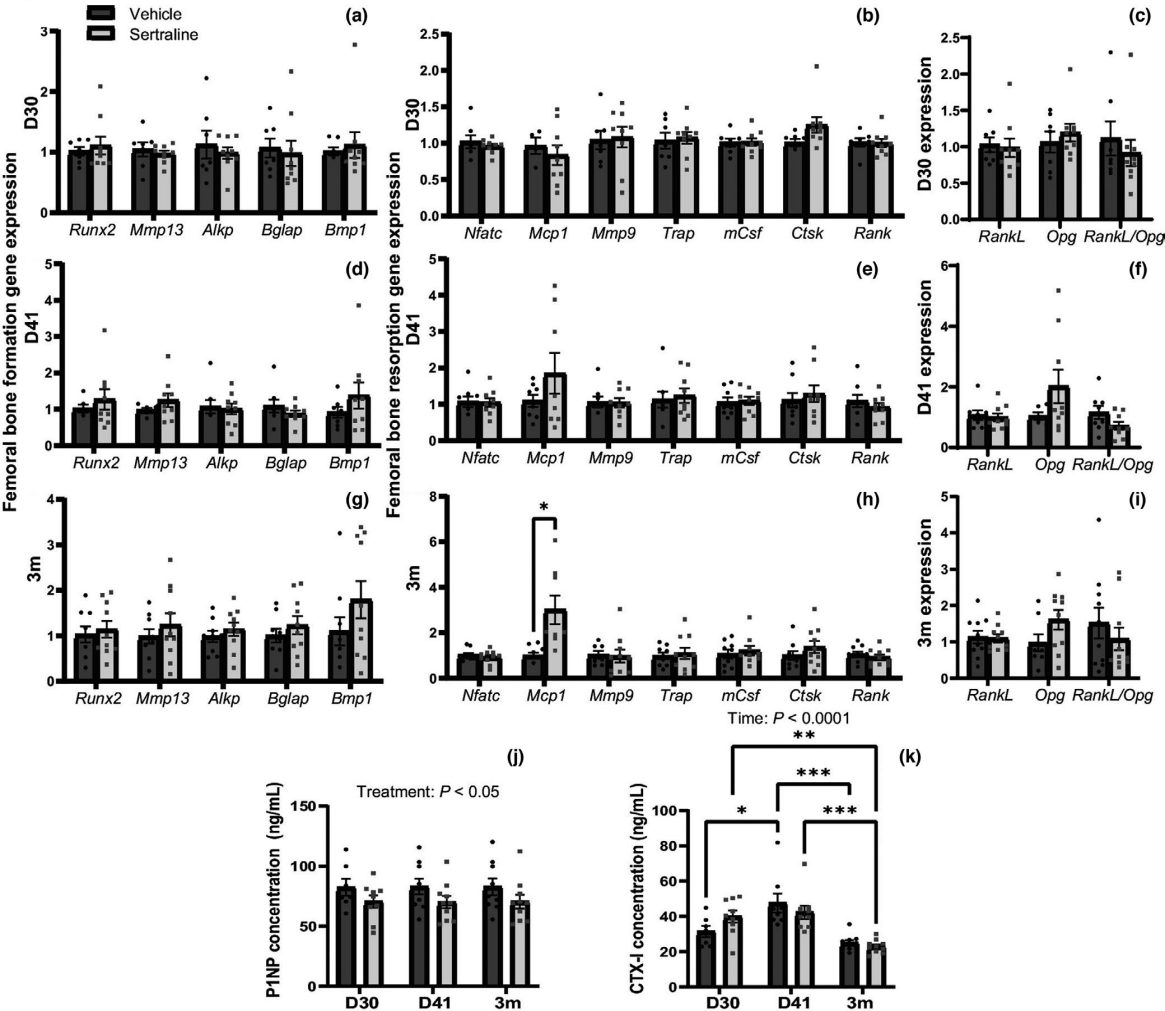
Peripartum sertraline reduced the circulating marker for bone formation but did not impact bone formation or resorption gene expression. Femurs were collected at D30, D41, or 3m post‐treatment and RNA was harvested from the entire femur using TRI‐reagent. RT‐qPCR was used to examine genes involved in bone formation and bone resorption at D30 (a–c), D41 (d–f), or 3m (g–i). Serum markers of bone formation, P1NP (j), and bone resorption, CTX (k), were measured. Dams were harvested at peak lactation (D30, treatment day 30) (*n* = 7 vehicle; *n* = 9 sertraline), weaning (D41, treatment day 41) (*n* = 9 vehicle; *n* = 9 sertraline), or were aged an additional 3 months after weaning (3m post‐treatment) (*n* = 10 vehicle; *n* = 10 sertraline). Data presented as mean ± SEM and analyzed using two‐way ANOVA for treatment and time or *t*‐test with multiple comparisons. *p < 0.05, **p < 0.01, and ***p < 0.001

Expression of key genes reflecting the activity of bone resorption (*Nfatc*, *Mcp1*, *Mmp9*, *Trap*, *mCSF*, *Ctsk*, and *Rank*) and formation (*Runx2*, *Mmp13*, *Alkp*, *Bglap*, and *Bmp1*) on D30, D41, and 3m post‐treatment (Figure 6a–i). An additional assessment of resorption was performed using the *RankL*/*Opg* ratio: the resorption gene (*RankL*) to the anti‐resorption (*Opg*). Bone formation genes were not impacted at any timepoint. We did not detect changes in femoral *Pthr1* or *Pthlh* gene expression (Figure S1). P1NP concentrations did not change throughout lactation, but sertraline overall decreased P1NP concentrations (*p* < 0.05, Figure 6j). Thus, systemic markers of bone turnover indicate a decrease in bone formation due to sertraline treatment, which is consistent with fluoxetine treatment (Weaver et al., 2018). There was no effect of sertraline on the *RankL*/*Opg* ratio was seen any timepoints. Similarly, bone resorption genes were not changed with sertraline‐treatment, except threefold increase in *Mcp1* expression, at 3m compared to vehicle‐treated dams (Figure 6h). Sertraline treatment did not alter overall bone resorption as measured by serum CTX‐I (Figure 6k). As expected, CTX‐I was higher in lactating mice (D30, D41) compared to 3m post‐lactation. Together, these data demonstrate that, unlike fluoxetine, sertraline does not negatively impact maternal bone structure, despite modest changes in bone turnover markers.

### 3.5 Maternal sertraline increases calcium trafficking in the duodenum at weaning and increases kidney calcium retention

**FIGURE 7:**
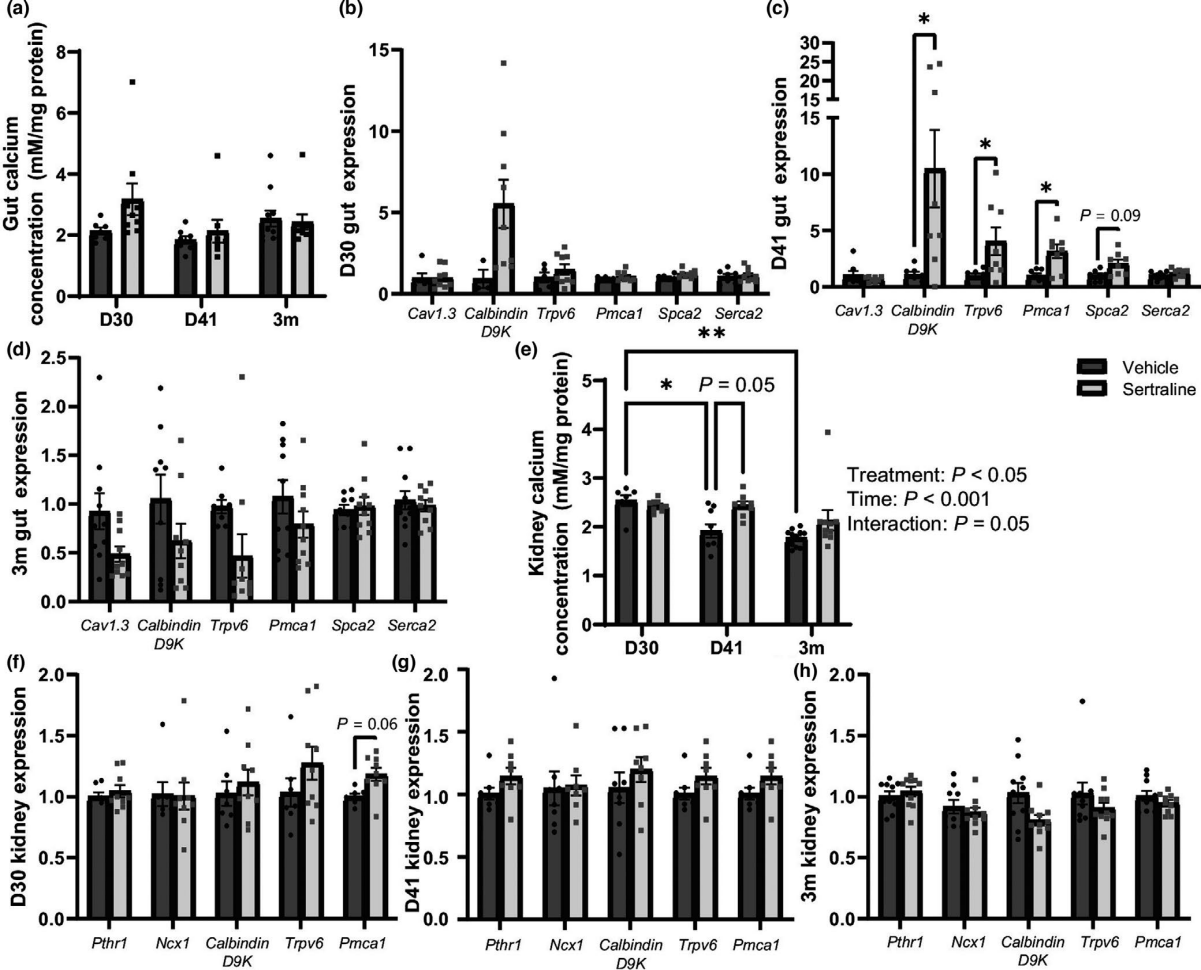
Sertraline increased kidney retention of calcium and increased duodenal calcium trafficking at weaning. Duodenal calcium concentration (mM/mg protein) was measured at D30, D41, and 3m (a). Calcium trafficking gene expression was measured in the duodenum at D30 (b), D41 (c), and 3m (d). Kidney calcium concentration (mM/mg protein) was measured at D30, D41, and 3m (e). Calcium trafficking gene expression was measured in the kidney at D30 (f), D41 (g), and 3m (h). Dams were harvested at peak lactation (D30, treatment day 30) (*n* = 7 vehicle; *n* = 9 sertraline), weaning (D41, treatment day 41) (*n* = 9 vehicle; *n* = 9 sertraline), or were aged an additional 3 months after weaning (3m post‐treatment) (*n* = 10 vehicle; *n* = 10 sertraline). Data presented as mean ± SEM and analyzed using two‐way ANOVA for treatment and time or *t*‐test with multiple comparisons. *p < 0.05, and **p < 0.01

Because the drop in circulating calcium was more drastic than the change in milk calcium alone, we explored other tissues that are involved in the regulation of calcium homeostasis. Duodenal calcium content was not affected by treatment or time (*p* > 0.05, Figure 7a). Additionally, we examined gene expression related to duodenal calcium trafficking (*Cav1.3*, *Trpv6*, *CalbindinD9K*, *Pmca1*, *Spca2*, and *Serca2*) on D30, D41, and 3m (Figure 7b–d). Increased *CalbindinD9K* expression was observed on D41 (*p* < 0.05). Furthermore, sertraline increased calcium trafficking genes, such as calcium absorption (*Trpv6*, *p* < 0.05) and utilization (*Pmca1*, *p* < 0.05) genes on D41 (Figure 7C). Kidney calcium content (Figure 7E) was increased by sertraline treatment (*p* < 0.05) with a tendency for sertraline‐mediated increase at D41 (*p* = 0.05), was altered by time (*p* < 0.001), with a tendency for an interaction between sertraline treatment and time (*p* = 0.05). We also measured expression of kidney calcium trafficking genes (*Pthr1*, *Ncx1*, *Calbindin D9K*, *Trpv6*, and *Pmca1*). Sertraline tended to increase *Pmca1* at D30 (*p* = 0.06), but no other differences in calcium trafficking genes occurred (Figure 7f–h). Although sertraline decreased circulating calcium, it was not due to decrease in duodenal absorption, though the kidney retained higher concentration of calcium.

### 3.6 Offspring are unaffected by maternal sertraline treatment

**FIGURE 8:**
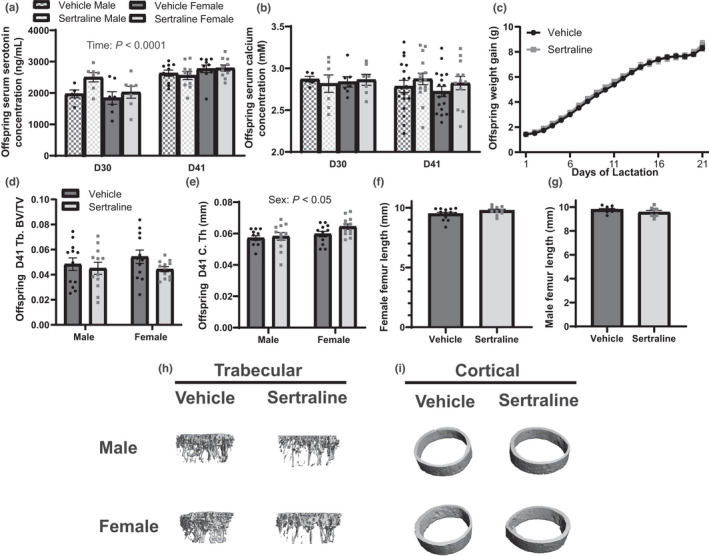
In utero and lactational exposure to sertraline does not impact offspring bone formation. Offspring was euthanized at postnatal day 10 (D30) or weaning, postnatal day 21 (D41). Serum serotonin concentration (ng/ml) was measured (a) and serum calcium concentration (mM) was measured (b). Offspring weight gain (g) was measured daily throughout lactation (c). D41 offspring was used for bone measurements to determine how sertraline exposure impacted offspring bone formation. Bone volume / total volume (BV/TV) was measured (d), cortical thickness (e), female femur length (f), and male femur length (g) were measured. Three‐dimensional reconstruction images of the trabecular bone (h) and cortical bone (i) are shown. Offspring were harvested at weaning (maternal treatment D41) for bone analysis (*n* = 12 vehicle, *n* = 12 female), and at peak lactation (maternal treatment D30) or weaning (D41) for serum analysis. For serum, all animals from the litter were pooled together separated by sex (D30 vehicle female *n* = 7 and male *n* = 6, D30 sertraline female *n* = 7 and male *n* = 7, D41 vehicle female *n* = 9 and male *n* = 9, D41 sertraline female *n* = 9 and male *n* = 9). Data presented as mean ± SEM and analyzed using two‐way ANOVA for treatment and time or Students’ *t*‐test

Previous results in offspring born to fluoxetine‐treated dams at weaning were shorter than those born to controls. Therefore, we examined if offspring born to sertraline‐treated dams were impacted by exposure during the peripartum period. Offspring were harvested at either postnatal day 10 (D30) or 21 (D41). Offspring sertraline concentrations were undetectable (data not shown). Additionally, pup circulating 5HT concentrations (Figure 8a) on D41 compared to D30 (*p* < 0.0001). Circulating calcium concentrations were unchanged due to sertraline exposure, sex, and timepoint (*p* > 0.05) (Figure 8b) and offspring weight gain was not affected by sertraline exposure (Figure 8c).

In utero and lactational exposure to fluoxetine impaired offspring trabecular and cortical bone, resulting in decreased trabecular BV/TV and increased cortical porosity (Weaver et al., 2019). We therefore examined if in utero and lactation exposure to sertraline similarly decreased offspring bone. Like the sertraline‐treated dams, trabecular BV/TV was unchanged in the sertraline‐exposed offspring (Figure 8d). Offspring trabecular total mineral density was significantly increased in sertraline‐exposed offspring (*p* < 0.05, Table 3). Cortical thickness was not altered in offspring exposed to sertraline but was significantly increased in the females compared to their male counterparts (*p* < 0.05, Figure 8e). Other trabecular and cortical parameters were unchanged due to sex or sertraline‐exposure (Tables 3 and 4, respectively). Furthermore, offspring femur length was unchanged in the male and female offspring exposed to sertraline (*p* > 0.05, Figure 8f,g). Together this data suggests that in utero and lactational exposure to sertraline does not impair offspring bone formation.

**TABLE 3:** Offspring femoral trabecular bone parameters evaluated by MicroCT

	Measurements				*p*‐value		
	Male		Female				
	VEH	SRT	VEH	SRT	Sex	Treatment	Interaction
Tb.N (1/mm)	3.8 ± 0.2	3.8 ± 0.2	3.7 ± 0.1	3.7 ± 0.09	0.68	0.91	0.84
Tb.Sp. (mm)	0.28 ± 0.01	0.28 ± 0.01	0.28 ± 0.009	0.27 ± 0.007	0.83	0.81	0.76
Tb.Th (mm)	0.023 ± 0.001	0.022 ± 0.001	0.024 ± 0.001	0.024 ± 0.001	0.074	0.17	0.83
Tissue BMD (mg Hg/cm^3^)	16.8 ± 5.0	16.9 ± 4.6	22.6 ± 5.3	11.4 ± 2.7	0.97	0.22	0.22
Tissue TMD (mg Hg/cm^3^)	998.1 ± 4.1	994.9 ± 3.3	1000.0 ± 4.8	1004.9 ± 3.3	0.33	0.010	0.96

Note

Offspring were harvested at weaning (maternal treatment D41) for bone analysis (*n* = 12 vehicle, *n* = 12 female). Data presented as mean ± SEM and analyzed using two‐way ANOVA for treatment and sex. *p* < 0.05 is considered significant and 0.1 < *p* < 0.05 is considered a tendency.

Abbreviations: BMD, bone mineral density; SRT, sertraline; Tb.N., trabecular number; Tb.Sp., trabecular spacing; Tb.Th., trabecular thickness; TMD, total mineral density; VEH, vehicle.

**TABLE 4:** Offspring femoral cortical bone parameters evaluated by MicroCT

	Measurements				*p*‐value		
	Male		Female				
	VEH	SRT	VEH	SRT	Sex	Treatment	Interaction
Periosteal area (mm^2^)	1.14 ± 0.05	1.16 ± 0.06	1.19 ± 0.07	1.12 ± 0.032	0.95	0.62	0.39
Periosteal porosity (mm)	0.81 ± 0.01	0.81 ± 0.01	0.81 ± 0.01	0.79 ± 0.01	0.11	0.18	0.12
Tissue BMD (mg Hg/cm^3^)	155.4 ± 5.6	147.1 ± 8.9	147.9 ± 7.3	160 ± 6.1	0.71	0.79	0.16
Tissue TMD (mg Hg/cm^3^)	881.6 ± 8.5	891.6 ± 10.6	892 ± 7.7	912.9 ± 10.1	0.099	0.11	0.57
Periosteal area (mm^2^)	1.14 ± 0.05	1.16 ± 0.06	1.19 ± 0.07	1.12 ± 0.03	0.95	0.62	0.39

Note

Offspring were harvested at weaning (maternal treatment D41) for bone analysis (*n* = 12 vehicle, *n* = 12 female). Data presented as mean ± SEM and analyzed using two‐way ANOVA for treatment and sex. *p* < 0.05 is considered significant and 0.1 < *p* < 0.05 is considered a tendency.

Abbreviations: BMD, bone mineral density; SRT, sertraline; TMD, total mineral density; VEH, vehicle.

### 3.7 Sertraline reduces circulating 5HT and calcium in nulliparous animals

**FIGURE 9:**
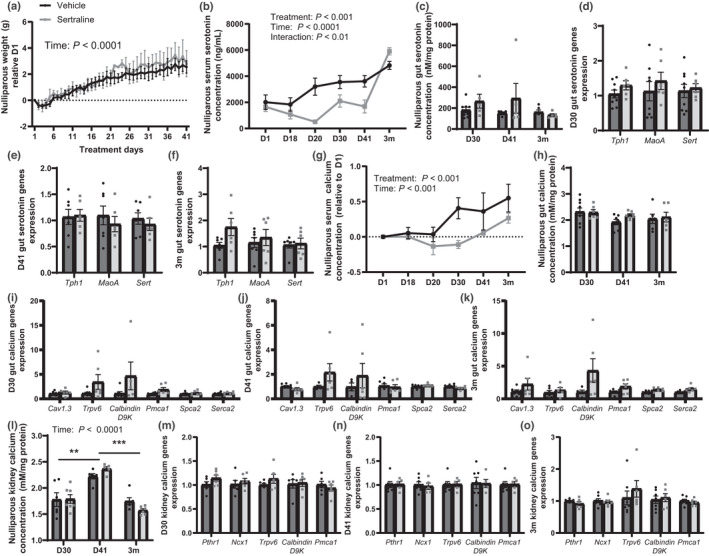
Nulliparous females treated with sertraline had reduced serum serotonin and calcium levels but did not impact kidney calcium retention. Age‐matched C57BL/6 nulliparous virgin female mice were treated with either vehicle (8.3% DMSO) or sertraline (10 mg/kg/day in 8.3% DMSO) for the same timeframe as the primiparous dams, as a bone control for pregnancy and lactation. Nulliparous weight (g) was measured daily, and the weight gain relative to start of treatment is shown (a). Serum serotonin concentration (ng/ml) was measured (b), duodenal serotonin (nM/mg protein) was measured (c), and duodenal serotonin metabolism gene expression was measured at D30 (d), D41 (e), and 3m post‐treatment (f). Serum calcium concentration (mM) was measured, calcium concentration was corrected to the baseline for each mouse (g). Duodenal calcium concentration (mM/mg protein) was measured (h) and duodenal calcium trafficking gene expression was measured at D30 (i), D41 (j), and 3m (k). Kidney calcium concentration (mM/mg protein) was measured (l) and kidney calcium trafficking gene expression was measured at D30 (m), D41 (n), and 3m (o). Nulliparous virgin mice were used as a control and harvested at treatment day 30 (*n* = 9 vehicle; *n* = 9 sertraline), treatment day 41 (*n* = 10 vehicle; *n* = 10 sertraline), or 3 months post‐treatment (*n* = 10 vehicle; *n* = 10 sertraline). Data presented as mean ± SEM and analyzed using two‐way ANOVA for treatment and time or *t*‐test with multiple comparisons. **p < 0.01, and ***p < 0.001

We additionally characterized the impact of sertraline on nulliparous animals (virgin females) to separate the effects of sertraline compared to lactation‐induced effects. Nulliparous females were exposed to the same treatment scheme as primiparous dams detailed in Figure 1a. Sertraline treatment did not impact nulliparous females weight gain (Figure 9a) but reduced circulating 5HT throughout the treatment period and was then unchanged by 3m post‐treatment (*p* < 0.001, Figure 9b). Like the primiparous dams, sertraline treatment did not impact duodenal 5HT content in nulliparous females (Figure 9c). We examined 5HT metabolism target genes (*Tph1*, *Maoa*, and *Sert*) at treatment D30, D41, and 3m post‐treatment (Figure 9d–f), finding no change with sertraline treatment.

Circulating calcium concentrations were reduced by sertraline treatment across the treatment period and remained so by 3m post‐treatment (*p* < 0.001, Figure 9g). Sertraline did not impact duodenal calcium content at any timepoint (Figure 9h), additionally, calcium trafficking gene expression (*Cav1.3*, *Trpv6*, *Calbindin D9K*, *Pmca1*, *Spca2*, and *Serca2*) was unchanged with sertraline treatment (Figure 9i–k). Sertraline did not impact kidney calcium content, but calcium concentrations changed overtime reaching peak concentrations at D41 (*p* < 0.0001, Figure 9l). Kidney gene expression of *Pthr1*, *Ncx1*, *Trpv6*, *Calbindin D9*, and *Pmca1* were unchanged at all timepoints (Figure 9m–o).

### 3.8 Sertraline does not impact nulliparous bone

**FIGURE 10:**
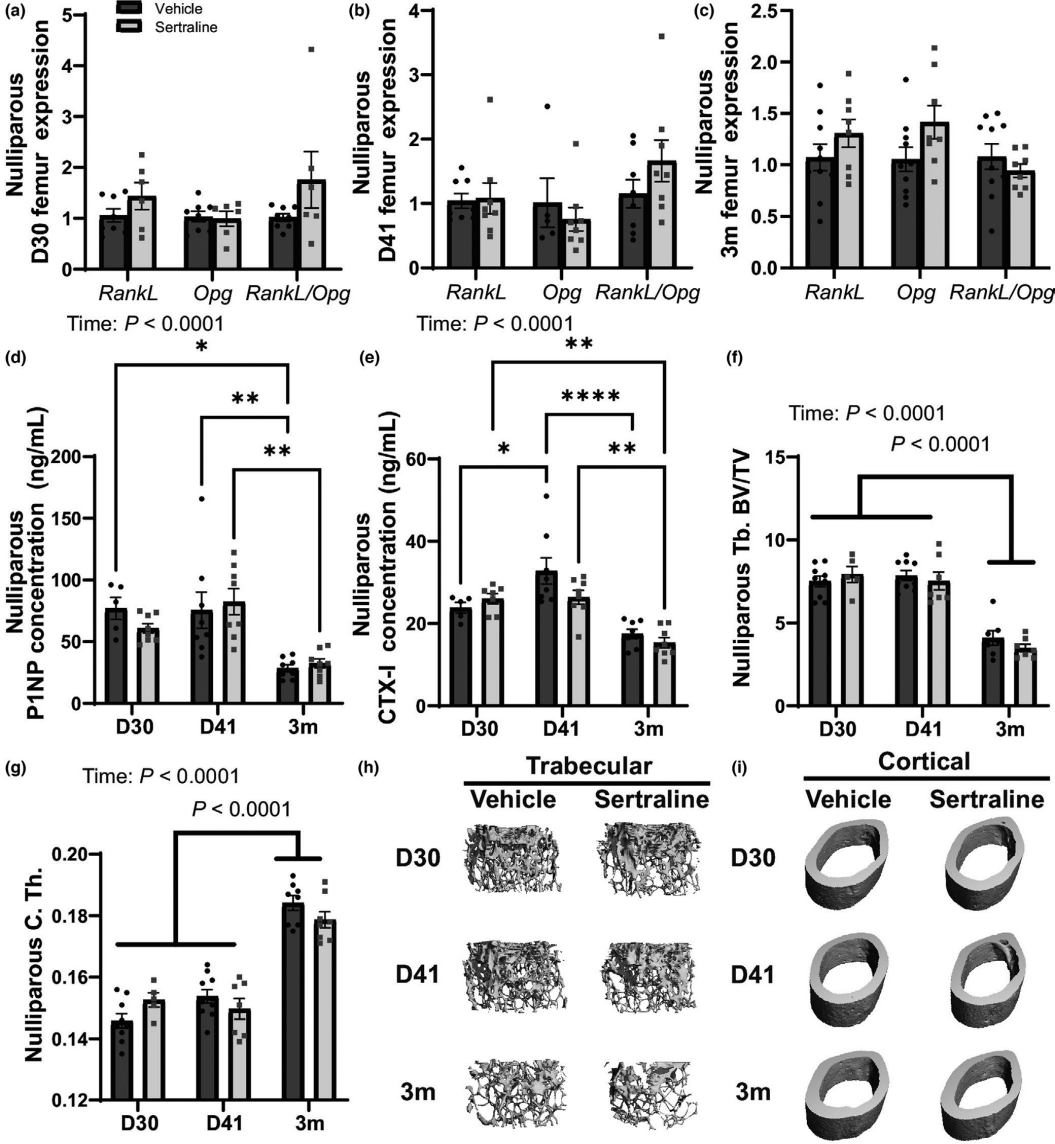
Sertraline did not compromise nulliparous female bone health. Nulliparous females were age‐matched to the primiparous females and euthanized on D30, D41, or 3m post‐treatment. Whole femurs were used for RNA extraction using TRI‐reagent and gene expression was measured with RT‐qPCR. Bone resorption versus anti‐resorption was measured with the *RankL*, *Opg*, and *RankL*/*Opg* ratio at D30 (a), D41 (b), and 3m (c). Serum bone formation, P1NP concentration (ng/ml) was measured (d), and bone resorption, CTX‐I concentration (ng/ml) was measured (e). Trabecular bone volume/total volume (BV/TV) was measured (f) and cortical thickness (g). Three‐dimensional reconstruction images of trabecular bone (h) and cortical bone (i) are shown. Nulliparous virgin mice were used as a control and harvested at treatment day 30 (*n* = 9 vehicle; *n* = 9 sertraline), treatment day 41 (*n* = 10 vehicle; *n* = 10 sertraline), or 3 months post‐treatment (*n* = 10 vehicle; *n* = 10 sertraline). Data presented as mean ± SEM and analyzed using two‐way ANOVA for treatment and time or *t*‐test with multiple comparisons. *p < 0.05, **p < 0.01, and ****p < 0.0001

We examined the impact of sertraline treatment on nulliparous bone to determine whether sertraline, in the absence of lactation, affects bone micro‐structure. We measured bone resorption genes (*Nfatc*, *Mcp1*, *Mmp9*, *Trap*, *mCsf*, *Ctsk*, and *Rank*) and bone formation genes (*Runx2*, *Mmp13*, *Alkp*, *Bglap*, and *Bmp1*) at D30, D41, and 3m post‐treatment (Figure S2), finding no change with sertraline treatment. The *RankL*/*Opg* ratio (Figure 10a–c) and *Pthlh* and *Pthr1* expression (Figure S1) were also unchanged with sertraline treatment. P1NP concentrations were unaffected by sertraline treatment, with decreased concentrations occurring at 3m post‐treatment (*p* < 0.0001, Figure 10d). CTX concentrations were similarly unchanged by sertraline treatment, but changed overtime, with all animals having decreased CTX concentrations by 3m post‐treatment (*p* < 0.0001, Figure 10e).

MicroCT analysis was used to determine structural changes with sertraline treatment in nulliparous animals. Similar to the primiparous dams, sertraline did not impact trabecular BV/TV, but BV/TV decreased significantly with age (3m post‐treatment) in all nulliparous females (*p* < 0.0001, Figure 10f). Cortical thickness was unchanged in sertraline‐treated nulliparous females but was increased by 3m post‐treatment (*p* < 0.0001, Figure 10g), similar to the primiparous dams. Sertraline treatment did not impact other trabecular (Table 5) or cortical parameters measured (Table 6), however, the nulliparous females displayed decreased trabecular number and increased trabecular spacing by 3m post‐treatment, similar to the primiparous dams. Together, these data demonstrate that sertraline did not compromise nulliparous female bone health.

**TABLE 5:** Nulliparous femoral trabecular bone parameters evaluated by MicroCT

	Measurements						*p*‐value		
	D30‐age match		D41‐age match		3m‐age match				
	VEH	SRT	VEH	SRT	VEH	SRT	Time	Treatment	Interaction
Tb. connective density	148.3 ± 8.8	164.0 ± 16.6	164.4 ± 6.4	152.7 ± 9.6	48.4 ± 2.8	47.3 ± 6.3	<0.0001	0.89	0.28
Tb.N. (1/mm)	3.9 ± 0.06	4.0 ± 0.2	3.8 ± 0.08	3.8 ± 0.06	2.8 ± 0.05	2.6 ± 0.08	<0.0001	0.84	0.39
Tb.Sp. (mm)	0.25 ± 0.004	0.25 ± 0.01	0.26 ± 0.005	0.26 ± 0.005	0.036 ± 0.007	0.38 ± 0.01	<0.0001	0.38	0.23
Tb.Th. (mm)	0.037 ± 0.001	0.037 ± 0.002	0.039 ± 0.001	0.038 ± 0.001	0.038 ± 0.002	0.037 ± 0.001	0.35	0.61	0.74
Tissue BMD (mg Hg/cm^3^)	63.8 ± 4.5	70.4 ± 6.2	69.6 ± 4.5	63.3 ± 7.05	18.7 ± 4.8	9.8 ± 3.4	<0.0001	0.50	0.31
Tissue TMD (mg Hg/cm^3^)	1012.6 ± 4.2	1019.0 ± 5.2	1021.5 ± 3.5	1012.1 ± 7.0	1040.7 ± 6.6	1032.9 ± 4.5	0.003	0.41	0.28

Note

Nulliparous virgin mice were used as a control and harvested at treatment day 30 (*n* = 9 vehicle; *n* = 5 sertraline), treatment day 41 (*n* = 9 vehicle; *n* = 7 sertraline), or 3 months post‐treatment (*n* = 7 vehicle; *n* = 7 sertraline). Data presented as mean ± SEM and analyzed using two‐way ANOVA for treatment and time. *p* < 0.05 is considered significant and 0.1 < *p* < 0.05 is considered a tendency.

Abbreviations: BMD, bone mineral density; SRT, sertraline; Tb.N., trabecular number; Tb.Sp., trabecular spacing; Tb.Th., trabecular thickness; TMD, total mineral density; VEH, vehicle.

**TABLE 6:** Nulliparous femoral cortical bone parameters evaluated by MicroCT

	Measurements						*p*‐value		
	D30‐age match		D41‐age match		3m‐age match				
	VEH	SRT	VEH	SRT	VEH	SRT	Time	Treatment	Interaction
Periosteal area (mm^2^)	1.77 ± 0.03	1.69 ± 0.07	1.73 ± 0.04	1.80 ± 0.06	1.82 ± 0.02	1.84 ± 0.04	0.07	0.85	0.24
Periosteal porosity (mm)	8.8 ± 0.09	8.5 ± 0.2	8.6 ± 0.1	8.9 ± 0.2	8.5 ± 0.06	8.6 ± 0.1	0.65	0.66	0.15
Tissue BMD (mg Hg/cm^3^)	461.4 ± 7.3	489.6 ± 6.2	493.8 ± 9.0	473.5 ± 13.9	582.5 ± 7.1	562.5 ± 8.8	<0.0001	0.61	0.03
Tissue TMD (mg Hg/cm^3^)	1201.9 ± 6.4	1205.7 ± 5.8	1218.4 ± 6.6	1214.4 ± 7.2	1287.7 ± 4.0	1275.8 ± 3.6	<0.0001	0.42	0.45

Note

Nulliparous virgin mice were used as a control and harvested at treatment day 30 (*n* = 9 vehicle; *n* = 5 sertraline), treatment day 41 (*n* = 10 vehicle; *n* = 7 sertraline), or 3 months post‐treatment (*n* = 8 vehicle; *n* = 8 sertraline). Data presented as mean ± SEM and analyzed using two‐way ANOVA for treatment and time. *p* < 0.05 is considered significant and 0.1 < *p* < 0.05 is considered a tendency.

Abbreviations: BMD, bone mineral density; SRT, sertraline; TMD, total mineral density; VEH, vehicle.

## 4 DISCUSSION

Contrary to our initial hypothesis that sertraline would affect bone like fluoxetine, we determined that this low dose of sertraline did not result in bone mass reductions in the mother, the offspring, or nulliparous females, indicating that it could be a safer therapeutic choice than fluoxetine for the skeleton with prolonged SSRI treatment in peripartal animals. Maternal trabecular bone was affected by time, but the effect of sertraline was not clear as there was only a tendency for a decreased trabecular BV/TV that only occurred at 3m post‐partum. Future studies could build on this initial study by investigating the effect of sertraline on maternal bone later in life and increasing the power, as these subtle effects may require additional animals to parse out the differences. All primiparous and nulliparous animals exhibited age‐associated skeletal changes by 3m post‐treatment. There is the possibility that these age‐associated changes were able to mask the potential mild sertraline‐lactation interaction.

Prolonged treatment with fluoxetine (6 weeks) in mice resulted in inhibited osteoclast differentiation and decreased bone formation, resulting in bone loss (Ortuno et al., 2016). Consistent with that study, sertraline reduced circulating P1NP concentrations, a marker indicating bone formation, suggesting a mild decrease in bone formation, although microCT parameters were largely unaffected. However, this low‐dose sertraline altered circulating calcium in an opposite manner to previous results in our lab using fluoxetine. These opposing effects of sertraline and fluoxetine on calcium and the skeleton suggest that although they are both within the SSRI class, that they have differential effects on the dam due to the pharmacological profiles of the drugs in addition to the varying doses (Ables & Baughman, 2003; DeVane et al., 2002; Sohel et al., 2021).

The dose used in this study (10 mg/kg/day) translates to ~50 mg in humans using the FDA body‐scale conversion (Nair & Jacob, 2016); which is the bottom of the range for recommended human prescribed doses of 50–200 mg/day. Marcus et al., interviewed 276 pregnant women finding 13% were currently taking an SSRI to treat depression, and the average dose of daily sertraline was 68.7 mg/day whereas the daily dose of fluoxetine was 23.3 mg/day (Marcus & Flynn, 2008). Conversely, our previous research with fluoxetine using 20 mg/kg/day approximated slightly above the high end (~90 mg) of the prescribed range of fluoxetine (20–80 mg/day) in humans (Nair & Jacob, 2016). Furthermore, sertraline has a relatively short half‐life of 22–36 h (DeVane et al., 2002). Unlike sertraline, fluoxetine's half‐life is 2–4 days, with the active metabolite, norfluoxetine, having a half‐life of 7–14 days (Sohel et al., 2021). Furthermore, fluoxetine inhibits the enzyme responsible for its metabolism, cytochrome 2D6, therefore, high doses or sustained treatment results in a further increase of the half‐life (Sanz et al., 2005; Sohel et al., 2021). This indicates a need for a head‐to‐head comparison with analogous doses to confirm the superior bone safety of sertraline compared to fluoxetine.

Beyond the functional outcome of bone loss due to SSRIs, we examined the intermediate signaling steps in this study to try to understand the mechanism by which SSRIs could be affecting bone. Outside the nervous system, SSRIs prevent 5HT from being transported into cells by uptake through SERT, resulting in an increase in intracellular (tissue) 5HT (Sheftel & Hernandez, 2020; Weaver et al., 2018). In this study, we report no change in mammary gland or duodenal 5HT concentration with sertraline treatment. However, we observed a significant decrease in circulating 5HT levels relative to baseline. Platelets lack the TPH1 enzyme required for 5HT synthesis resulting in decreased platelet 5HT with SSRI exposure (Bismuth‐Evenzal et al., 2012; Holck et al., 2019; Rossum et al., 2020). Together, these findings suggest that the dose of sertraline used is having a systemic physiological response, although there may not be an increase in tissue 5HT due to the shorter half‐life of the drug. One limitation of this study is the fact that the dams must undergo a 6‐h fast prior to collecting blood since food intake can affect the abundance of bone resorption markers in the circulation (Clowes et al., 2002). The 6‐h fast between dosing and blood collection, could result in missing the window of highest sertraline potency. We suspect the sertraline dose used in this study causes a transient 5HT response due to the short half‐life and lower dose rather than the prolonged 5HT response observed with higher dose fluoxetine treatment.

During lactation, calcium homeostasis is regulated by PTHrP, which is synthesized in the mammary gland and secreted. Upon entering circulation, PTHrP can act on the bone to liberate calcium for milk synthesis through binding to the parathyroid hormone receptor 1 (PTHR1). Previously, we have demonstrated that mammary‐derived 5HT regulates PTHrP through autocrine/paracrine 5HT2B receptor signaling, as well as intracellular epigenetic alterations of sonic hedgehog, and through the molecular process of serotonylation (Hernandez et al., 2012; Laporta, Keil, Weaver, et al., 2014; Sheftel & Hernandez, 2020). PTHrP is also regulated by the calcium sensing receptor, which works in a negative feedback loop with PTHrP (Mamillapalli & Wysolmerski, 2010). In previous in vivo and in vitro studies, fluoxetine treatment results in an increase in tissue 5HT and subsequent increase in mammary PTHrP (*Pthlh* gene) (Hernandez et al., 2012; Sheftel & Hernandez, 2020) however, this was not the case with sertraline in this study. Thus, the observed bone loss with fluoxetine but not with sertraline could be explained by the presence or absence of sustained 5HT‐induced PTHrP signaling with different SSRIs.

In a non‐lactating state, 95% of 5HT is synthesized in the gut, and calcium homeostasis in a non‐lactating state is maintained through the gut, kidney, bone, and parathyroid gland (Banskota et al., 2019; Ramasamy, 2006). We examined how sertraline affects peripheral 5HT and calcium homeostasis through duodenal and kidney calcium trafficking. Sertraline treatment reduced circulating calcium concentrations, increased kidney calcium concentrations, and increased intestinal gene expression of *Calbindin‐D9k*, *Trpv6*, and *Pmca1* (calcium trafficking proteins) on D41 in the primiparous group, resulting in a restoration of circulating calcium to vehicle‐treated levels by 3m post‐treatment. This suggests that in the sertraline‐treated dams, the maternal body is responding to decreased circulating calcium by increasing the calcium absorption in the duodenum while decreasing the excretion in kidneys. Interestingly, over time, vehicle‐treated dams experienced a decrease in kidney calcium content, whereas the sertraline‐treated dams remained constant. During lactation, intestinal *Pmca1* and *Calbindin‐D9k* increases (Zhu et al., 1998) however, the maternal body primarily responds to the lactation‐associated calcium demand by mobilizing calcium from the skeleton while reducing calcium excretion from the kidney (Kovacs & Kronenberg, 1997).

One limitation of this study is that we did not collect urine samples, therefore, we cannot confirm that sertraline further decreased calcium excretion, but suspect is based on the kidney calcium content. The nulliparous females treated with sertraline had a sustained reduction of circulating calcium through 3m post‐treatment, which was accompanied by no alterations in kidney calcium content. This suggests that sertraline may be influencing calcium homeostasis differently in lactating compared to non‐lactating mice. Future studies should aim to differentiate how SSRIs impact the calcemic hormones (PTH, calcitriol, and calcitonin), particularly in the non‐lactating bone control mice. Particularly, because calcitriol increases intestinal calcium absorption and kidney calcium reabsorption (Horst et al., 1994).

5HT has an important autocrine/paracrine role during lactation on involution of the mammary gland due 5HT’s interaction with the 5HT receptor 7 (Pai et al., 2015) in addition to its role in inducing PTHrP. 5HT and SSRIs, particularly fluoxetine, accelerate involution by disassembly of tight junctions in a biphasic manner, reducing milk yield (Hernandez et al., 2011; Marshall et al., 2010; Pai et al., 2015; Pai & Horseman, 2008). Herein, we reported sertraline treatment resulted in a significant increase in gene expression of *5htr7* in the mammary gland. By D41, ZO‐1 immunofluorescent staining revealed a significant reduction in both the vehicle‐ and sertraline‐treated dams, with sertraline dams having undetectable levels of ZO‐1 as observed by immunofluorescence. This suggests that both control and sertraline mammary glands were undergoing involution, but sertraline treatment may result in a mildly faster involution. This could possibly be due to signaling via the *5htr7*, however this needs to be examined more closely. Additional work is needed to confirm whether sertraline, like fluoxetine, accelerates involution. This finding could be important for women using SSRIs during lactation and possible impacts on maintenance of milk production for their babies (Marshall et al., 2010).

Sertraline treatment results in the lowest umbilical cord serum concentrations among SSRIs, suggesting a lower fetal exposure, and sertraline appears to have the lowest concentration of crossover into the breast milk during lactation compared to other SSRIs (Gentile, 2005; Hendrick et al., 2003; Meltzer‐Brody, 2011; Payne, 2007). One study examining the excretion of fluoxetine and the metabolite, norfluoxetine, in human breast milk, found that infants were ingesting approximately 10.8% of the maternal dose (Taddio et al., 1996). However, another study examining excretion of sertraline and the metabolite, desmethylsertraline, in human breast milk, found that infants were ingesting approximately 0.54% of the maternal dose (Stowe et al., 1997). This suggests that the offspring of mothers on sertraline have a lower daily SSRI exposure through the milk compared to fluoxetine. Our data supports this as we were unable to detect sertraline concentrations in the offspring. Furthermore, the pups had similar concentrations of calcium and 5HT in their serum, suggesting that sertraline did not impact calcium homeostasis or 5HT signaling in the pups through exposure to SSRI via milk. Sertraline treatment did not impact mammary calcium content or transporters in the primiparous dams. However, milk calcium content tended to decrease in the sertraline‐treated dams. Offspring serum calcium concentrations were unchanged throughout lactation and were unaffected by sertraline treatment, suggesting that although milk calcium content was mildly decreased, the offspring still received adequate calcium from the diet. Offspring exposed to fluoxetine during pregnancy and lactation had shorter long bones and a decreased head circumference (Weaver et al., 2019), but we did not observe a change in long bone length with sertraline treatment. This suggests in utero and lactational exposure to sertraline does not impact offspring bone formation.

In summary, sertraline treatment caused a reduction in circulating calcium concentrations, which led to compensatory increases in kidney and duodenal calcium uptake. More importantly, sertraline treatment did not exacerbate or lead to a sustained reduction of maternal BMD, and sertraline did not impact offspring bone formation. Further research is needed to assess high‐dose sertraline on maternal and offspring BMD to directly compare its effects with high‐dose fluoxetine. However, sertraline treatment may result in a more rapid mammary gland involution, given it increased alveoli number and reduced ZO‐1, a tight junction protein, on day 21 of lactation. Further research is needed to confirm whether sertraline results in impaired breastfeeding durations. Together, this research adds to the evidence base suggesting that sertraline is a better choice of antidepressant during the peripartum period for preservation of maternal bone density as well as for offspring bone formation but may impact the duration of lactation.

## CONFLICT OF INTEREST

The authors declare no conflict of interest regarding this manuscript.

## AUTHOR CONTRIBUTIONS

Celeste M. Sheftel, Luma C. Sartori, Emily R. Hunt, Robbie S. J. Manuel, Autumn M. Bell, Rafael R. Domingues, Lella A. Wake, Brandon R. Scharpf, Chad M. Vezina, Julia F. Charles, and Laura L. Hernandez, collected samples and analyzed data. Celeste M. Sheftel, Julia F. Charles, and Laura L. Hernandez, designed this research. Celeste M. Sheftel, Julia F. Charles, and Laura L. Hernandez wrote the initial draft. All authors read and proved the final manuscript.

## Supporting information



Table S1

Figure S1

Figure S2

## References

[phy215204-bib-0001] Ables, A. Z. , & Baughman, O. L. III (2003). Antidepressants: Update on new agents and indications. American Family Physician, 67, 547–554. 12588077

[phy215204-bib-0002] Andrade, S. E. , Raebel, M. A. , Brown, J. , Lane, K. , Livingston, J. , Boudreau, D. , Rolnick, S. J. , Roblin, D. , Smith, D. H. , Willy, M. E. , Staffa, J. A. , & Platt, R. (2008). Use of antidepressant medications during pregnancy: A multisite study. American Journal of Obstetrics and Gynecology, 198(194), e191–e195. 10.1016/j.ajog.2007.07.036 17905176

[phy215204-bib-0003] Banskota, S. , Ghia, J. E. , & Khan, W. I. (2019). Serotonin in the gut: Blessing or a curse. Biochimie, 161, 56–64. 29909048 10.1016/j.biochi.2018.06.008

[phy215204-bib-0004] Bismuth‐Evenzal, Y. , Gonopolsky, Y. , Gurwitz, D. , Iancu, I. , Weizman, A. , & Rehavi, M. (2012). Decreased serotonin content and reduced agonist‐induced aggregation in platelets of patients chronically medicated with SSRI drugs. Journal of Affective Disorders, 136, 99–103. 10.1016/j.jad.2011.08.013 21893349

[phy215204-bib-0005] Bjornerem, A. , Ghasem‐Zadeh, A. , Wang, X. , Bui, M. , Walker, S. P. , Zebaze, R. , & Seeman, E. (2017). Irreversible deterioration of cortical and trabecular microstructure associated with breastfeeding. Journal of Bone and Mineral Research, 32, 681–687. 10.1002/jbmr.3018 27736021

[phy215204-bib-0006] Bonnet, N. , Bernard, P. , Beaupied, H. , Bizot, J. C. , Trovero, F. , Courteix, D. , & Benhamou, C. L. (2007). Various effects of antidepressant drugs on bone microarchitectecture, mechanical properties and bone remodeling. Toxicology and Applied Pharmacology, 221, 111–118. 10.1016/j.taap.2007.02.005 17383703

[phy215204-bib-0007] Bouxsein, M. L. , Boyd, S. K. , Christiansen, B. A. , Guldberg, R. E. , Jepsen, K. J. , & Muller, R. (2010). Guidelines for assessment of bone microstructure in rodents using micro‐computed tomography. Journal of Bone and Mineral Research, 25, 1468–1486. 10.1002/jbmr.141 20533309

[phy215204-bib-0008] Clowes, J. A. , Hannon, R. A. , Yap, T. S. , Hoyle, N. R. , Blumsohn, A. , & Eastell, R. (2002). Effect of feeding on bone turnover markers and its impact on biological variability of measurements. Bone, 30, 886–890. 10.1016/S8756-3282(02)00728-7 12052458

[phy215204-bib-0009] Cooper, W. O. , Willy, M. E. , Pont, S. J. , & Ray, W. A. (2007). Increasing use of antidepressants in pregnancy. American Journal of Obstetrics and Gynecology, 196(544), e541–e545. 10.1016/j.ajog.2007.01.033 17547888

[phy215204-bib-0010] Davanzo, R. , Copertino, M. , De Cunto, A. , Minen, F. , & Amaddeo, A. (2011). Antidepressant drugs and breastfeeding: A review of the literature. Breastfeeding Medicine, 6, 89–98. 20958101 10.1089/bfm.2010.0019

[phy215204-bib-0011] DeVane, C. L. , Liston, H. L. , & Markowitz, J. S. (2002). Clinical pharmacokinetics of sertraline. Clinical Pharmacokinetics, 41, 1247–1266. 10.2165/00003088-200241150-00002 12452737

[phy215204-bib-0012] Domingues, R. R. , Fricke, H. P. , Sheftel, C. M. , Bell, A. M. , Sartori, L. C. , Manuel, R. S. J. , Krajco, C. J. , Wiltbank, M. C. , & Hernandez, L. L. (2022). Effect of low and high doses of two selective serotonin reuptake inhibitors on pregnancy outcomes and neonatal mortality. Toxics, 10, 11. 10.3390/toxics10010011 35051053 PMC8780128

[phy215204-bib-0013] Gavin, N. I. , Gaynes, B. N. , Lohr, K. N. , Meltzer‐Brody, S. , Gartlehner, G. , & Swinson, T. (2005). Perinatal depression: A systematic review of prevalence and incidence. Obstetrics and Gynecology, 106, 1071–1083. 16260528 10.1097/01.AOG.0000183597.31630.db

[phy215204-bib-0014] Gentile, S. (2005). The safety of newer antidepressants in pregnancy and breastfeeding. Drug Safety, 28, 137–152. 10.2165/00002018-200528020-00005 15691224

[phy215204-bib-0015] Hendrick, V. , Stowe, Z. N. , Altshuler, L. L. , Hwang, S. , Lee, E. , & Haynes, D. (2003). Placental passage of antidepressant medications. American Journal of Psychiatry, 160, 993–996. 10.1176/appi.ajp.160.5.993 12727706

[phy215204-bib-0016] Hernandez, L. L. , Collier, J. L. , Vomachka, A. J. , Collier, R. J. , & Horseman, N. D. (2011). Suppression of lactation and acceleration of involution in the bovine mammary gland by a selective serotonin reuptake inhibitor. Journal of Endocrinology, 209, 45–54. 10.1530/JOE-10-0452 21307120

[phy215204-bib-0017] Hernandez, L. L. , Gregerson, K. A. , & Horseman, N. D. (2012). Mammary gland serotonin regulates parathyroid hormone‐related protein and other bone‐related signals. American Journal of Physiology. Endocrinology and Metabolism, 302, E1009–E1015. 10.1152/ajpendo.00666.2011 22318950 PMC3774078

[phy215204-bib-0018] Holck, A. , Wolkowitz, O. M. , Mellon, S. H. , Reus, V. I. , Nelson, J. C. , Westrin, A. , & Lindqvist, D. (2019). Plasma serotonin levels are associated with antidepressant response to SSRIs. Journal of Affective Disorders, 250, 65–70. 10.1016/j.jad.2019.02.063 30831543 PMC6699768

[phy215204-bib-0019] Horst, R. L. , Goff, J. P. , & Reinhardt, T. A. (1994). Calcium and vitamin D metabolism in the dairy cow. Journal of Dairy Science, 77, 1936–1951. 10.3168/jds.S0022-0302(94)77140-X 7929956

[phy215204-bib-0020] Howie, R. N. , Herberg, S. , Durham, E. , Grey, Z. , Bennfors, G. , Elsalanty, M. , LaRue, A. C. , Hill, W. D. , & Cray, J. J. (2018). Selective serotonin re‐uptake inhibitor sertraline inhibits bone healing in a calvarial defect model. International Journal of Oral Science, 10, 25. 10.1038/s41368-018-0026-x 30174329 PMC6119683

[phy215204-bib-0021] Hwang, I. R. , Choi, Y. K. , Lee, W. K. , Kim, J. G. , Lee, I. K. , Kim, S. W. , & Park, K. G. (2016). Association between prolonged breastfeeding and bone mineral density and osteoporosis in postmenopausal women: KNHANES 2010–2011. Osteoporosis International, 27, 257–265. 26373982 10.1007/s00198-015-3292-x

[phy215204-bib-0022] Kim, H. J. , Kwon, H. , Oh, S. W. , Lee, C. M. , Joh, H. K. , Kim, Y. , Um, Y. J. , & Ahn, S. H. (2015). Breast feeding is associated with postmenopausal bone loss: Findings from the Korea National Health and Nutrition Examination Survey. Korean Journal of Family Medicine, 36, 216–220. 26435811 10.4082/kjfm.2015.36.5.216 PMC4591386

[phy215204-bib-0023] Koren, G. , & Nordeng, H. (2012). Antidepressant use during pregnancy: The benefit‐risk ratio. American Journal of Obstetrics and Gynecology, 207, 157–163. 22425404 10.1016/j.ajog.2012.02.009

[phy215204-bib-0024] Kovacs, C. S. (2016). Maternal mineral and bone metabolism during pregnancy, lactation, and post‐weaning recovery. Physiological Reviews, 96, 449–547. 10.1152/physrev.00027.2015 26887676

[phy215204-bib-0025] Kovacs, C. S. , & Kronenberg, H. M. (1997). Maternal‐fetal calcium and bone metabolism during pregnancy, puerperium, and lactation. Endocrine Reviews, 18, 832–872. 10.1210/edrv.18.6.0319 9408745

[phy215204-bib-0026] Kumar, M. , Wadhwa, R. , Kothari, P. , Trivedi, R. , & Vohora, D. (2018). Differential effects of serotonin reuptake inhibitors fluoxetine and escitalopram on bone markers and microarchitecture in Wistar rats. European Journal of Pharmacology, 825, 57–62. 10.1016/j.ejphar.2018.02.026 29470959

[phy215204-bib-0027] Laporta, J. , Keil, K. P. , Vezina, C. M. , & Hernandez, L. L. (2014). Peripheral serotonin regulates maternal calcium trafficking in mammary epithelial cells during lactation in mice. PLoS One, 9, e110190. 10.1371/journal.pone.0110190 25299122 PMC4192539

[phy215204-bib-0028] Laporta, J. , Keil, K. P. , Weaver, S. R. , Cronick, C. M. , Prichard, A. P. , Crenshaw, T. D. , Heyne, G. W. , Vezina, C. M. , Lipinski, R. J. , & Hernandez, L. L. (2014). Serotonin regulates calcium homeostasis in lactation by epigenetic activation of hedgehog signaling. Molecular Endocrinology, 28, 1866–1874. 10.1210/me.2014-1204 25192038 PMC4213360

[phy215204-bib-0029] Laporta, J. , Peters, T. L. , Weaver, S. R. , Merriman, K. E. , & Hernandez, L. L. (2013). Feeding 5‐hydroxy‐l‐tryptophan during the transition from pregnancy to lactation increases calcium mobilization from bone in rats. Domestic Animal Endocrinology, 44, 176–184. 10.1016/j.domaniend.2013.01.005 23433710

[phy215204-bib-0030] Liu, X. S. , Ardeshirpour, L. , VanHouten, J. N. , Shane, E. , & Wysolmerski, J. J. (2012). Site‐specific changes in bone microarchitecture, mineralization, and stiffness during lactation and after weaning in mice. Journal of Bone and Mineral Research, 27, 865–875. 10.1002/jbmr.1503 22189918

[phy215204-bib-0031] Mamillapalli, R. , & Wysolmerski, J. (2010). The calcium‐sensing receptor couples to Galpha(s) and regulates PTHrP and ACTH secretion in pituitary cells. Journal of Endocrinology, 204, 287–297. 10.1677/JOE-09-0183 PMC3777408 20032198

[phy215204-bib-0032] Marcus, S. M. , & Flynn, H. A. (2008). Depression, antidepressant medication, and functioning outcomes among pregnant women. International Journal of Gynaecology and Obstetrics, 100, 248–251. 10.1016/j.ijgo.2007.09.016 18005968

[phy215204-bib-0033] Marcus, S. M. , Flynn, H. A. , Blow, F. C. , & Barry, K. L. (2003). Depressive symptoms among pregnant women screened in obstetrics settings. Journal of Women's Health, 12, 373–380. 10.1089/154099903765448880 12804344

[phy215204-bib-0034] Marshall, A. M. , Hernandez, L. L. , & Horseman, N. D. (2014). Serotonin and serotonin transport in the regulation of lactation. Journal of Mammary Gland Biology and Neoplasia, 19, 139–146. 10.1007/s10911-013-9304-6 24136337

[phy215204-bib-0035] Marshall, A. M. , Nommsen‐Rivers, L. A. , Hernandez, L. L. , Dewey, K. G. , Chantry, C. J. , Gregerson, K. A. , & Horseman, N. D. (2010). Serotonin transport and metabolism in the mammary gland modulates secretory activation and involution. Journal of Clinical Endocrinology and Metabolism, 95, 837–846. 10.1210/jc.2009-1575 19965920 PMC2840848

[phy215204-bib-0036] Martin, A. M. , Young, R. L. , Leong, L. , Rogers, G. B. , Spencer, N. J. , Jessup, C. F. , & Keating, D. J. (2017). The diverse metabolic roles of peripheral serotonin. Endocrinology, 158, 1049–1063. 10.1210/en.2016-1839 28323941

[phy215204-bib-0037] Meltzer‐Brody, S. (2011). New insights into perinatal depression: Pathogenesis and treatment during pregnancy and postpartum. Dialogues in Clinical Neuroscience, 13, 89–100. 21485749 10.31887/DCNS.2011.13.1/smbrody PMC3181972

[phy215204-bib-0038] Mgodi, N. M. , Kelly, C. , Gati, B. , Greenspan, S. , Dai, J. Y. , Bragg, V. , Livant, E. , Piper, J. M. , Nakabiito, C. , Magure, T. , Marrazzo, J. M. , Chirenje, Z. M. , Riddler, S. A. ; Team M‐BP . (2015). Factors associated with bone mineral density in healthy African women. Archives of Osteoporosis, 10, 206. 10.1007/s11657-015-0206-7 25680424 PMC4564062

[phy215204-bib-0039] Miyamoto, T. , Miyakoshi, K. , Sato, Y. , Kasuga, Y. , Ikenoue, S. , Miyamoto, K. , Nishiwaki, Y. , Tanaka, M. , Nakamura, M. , & Matsumoto, M. (2019). Changes in bone metabolic profile associated with pregnancy or lactation. Scientific Reports, 9, 6787. 10.1038/s41598-019-43049-1 31086225 PMC6513862

[phy215204-bib-0040] Nair, A. B. , & Jacob, S. (2016). A simple practice guide for dose conversion between animals and human. Journal of Basic and Clinical Pharmacy, 7, 27–31. 10.4103/0976-0105.177703 27057123 PMC4804402

[phy215204-bib-0041] Okyay, D. O. , Okyay, E. , Dogan, E. , Kurtulmus, S. , Acet, F. , & Taner, C. E. (2013). Prolonged breast‐feeding is an independent risk factor for postmenopausal osteoporosis. Maturitas, 74, 270–275. 10.1016/j.maturitas.2012.12.014 23352271

[phy215204-bib-0042] Ortuno, M. J. , Robinson, S. T. , Subramanyam, P. , Paone, R. , Huang, Y. Y. , Guo, X. E. , Colecraft, H. M. , Mann, J. J. , & Ducy, P. (2016). Serotonin‐reuptake inhibitors act centrally to cause bone loss in mice by counteracting a local anti‐resorptive effect. Nature Medicine, 22, 1170–1179. 10.1038/nm.4166 PMC5053870 27595322

[phy215204-bib-0043] Pai, V. P. , Hernandez, L. L. , Stull, M. A. , & Horseman, N. D. (2015). The type 7 serotonin receptor, 5‐HT 7, is essential in the mammary gland for regulation of mammary epithelial structure and function. BioMed Research International, 2015, 364746. 25664318 10.1155/2015/364746 PMC4312625

[phy215204-bib-0044] Pai, V. P. , & Horseman, N. D. (2008). Biphasic regulation of mammary epithelial resistance by serotonin through activation of multiple pathways. Journal of Biological Chemistry, 283, 30901–30910. 10.1074/jbc.M802476200 PMC2576527 18782769

[phy215204-bib-0045] Payne, J. L. (2007). Antidepressant use in the postpartum period: Practical considerations. American Journal of Psychiatry, 164, 1329–1332. 10.1176/appi.ajp.2007.07030390 17728416

[phy215204-bib-0046] Ramasamy, I. (2006). Recent advances in physiological calcium homeostasis. Clinical Chemistry and Laboratory Medicine, 44, 237–273. 10.1515/CCLM.2006.046 16519596

[phy215204-bib-0047] Rapport, M. M. , Green, A. A. , & Page, I. H. (1948). Serum vasoconstrictor, serotonin; isolation and characterization. Journal of Biological Chemistry, 176, 1243–1251. 10.1016/S0021-9258(18)57137-4 18100415

[phy215204-bib-0048] Ritchie, L. D. , Fung, E. B. , Halloran, B. P. , Turnlund, J. R. , Van Loan, M. D. , Cann, C. E. , & King, J. C. (1998). A longitudinal study of calcium homeostasis during human pregnancy and lactation and after resumption of menses. American Journal of Clinical Nutrition, 67, 693–701. 10.1093/ajcn/67.4.693 9537616

[phy215204-bib-0049] Rojano‐Mejia, D. , Aguilar‐Madrid, G. , Lopez‐Medina, G. , Cortes‐Espinosa, L. , Hernandez‐Chiu, M. C. , Canto‐Cetina, T. , Vergara‐Lopez, A. , Coral‐Vazquez, R. M. , & Canto, P. (2011). Risk factors and impact on bone mineral density in postmenopausal Mexican mestizo women. Menopause, 18, 302–306. 10.1097/gme.0b013e3181f2d3fb 20881651

[phy215204-bib-0050] Ryan, B. A. , & Kovacs, C. S. (2019). The puzzle of lactational bone physiology: Osteocytes masquerade as osteoclasts and osteoblasts. Journal of Clinical Investigation, 129, 3041–3044. 10.1172/JCI130640 PMC6668815 31232705

[phy215204-bib-0051] Sanz, E. J. , De‐las‐Cuevas, C. , Kiuru, A. , Bate, A. , & Edwards, R. (2005). Selective serotonin reuptake inhibitors in pregnant women and neonatal withdrawal syndrome: A database analysis. Lancet, 365, 482–487. 15705457 10.1016/S0140-6736(05)17865-9

[phy215204-bib-0052] Sheftel, C. M. , & Hernandez, L. L. (2020). Serotonin stimulated parathyroid hormone related protein induction in the mammary epithelia by transglutaminase‐dependent serotonylation. PLoS One, 15, e0241192. 10.1371/journal.pone.0241192 33095824 PMC7584195

[phy215204-bib-0053] Sohel, A. J. , Shutter, M. C. , & Molla, M. (2021). Fluoxetine. In StatPearls [Internet]. Treasure Island, FL: StatPearls Publishing. https://www.ncbi.nlm.nih.gov/books/NBK459223/ 29083803

[phy215204-bib-0054] Sowers, M. , Corton, G. , Shapiro, B. , Jannausch, M. L. , Crutchfield, M. , Smith, M. L. , Randolph, J. F. , & Hollis, B. (1993). Changes in bone density with lactation. JAMA, 269, 3130–3135. 10.1001/jama.269.24.3130 8505816

[phy215204-bib-0055] Stowe, Z. N. , Owens, M. J. , Landry, J. C. , Kilts, C. D. , Ely, T. , Llewellyn, A. , & Nemeroff, C. B. (1997). Sertraline and desmethylsertraline in human breast milk and nursing infants. American Journal of Psychiatry, 154, 1255–1260. 10.1176/ajp.154.9.1255 9286185

[phy215204-bib-0056] Taddio, A. , Ito, S. , & Koren, G. (1996). Excretion of fluoxetine and its metabolite, norfluoxetine, in human breast milk. Journal of Clinical Pharmacology, 36, 42–47. 10.1002/j.1552-4604.1996.tb04150.x 8932542

[phy215204-bib-0057] Tsapakis, E. M. , Gamie, Z. , Tran, G. T. , Adshead, S. , Lampard, A. , Mantalaris, A. , & Tsiridis, E. (2012). The adverse skeletal effects of selective serotonin reuptake inhibitors. European Psychiatry, 27, 156–169. 10.1016/j.eurpsy.2010.10.006 21295451

[phy215204-bib-0058] van Rossum, H. H. , Spruit, J. , Korse, C. M. , de Vries, F. E. , & Tesselaar, M. E. T. (2020). Antidepressant use limits serotonin as a marker for neuroendocrine tumor disease activity by lowering of circulating serotonin concentrations. Clinical Chemistry and Laboratory Medicine, 58, e241–e243. 10.1515/cclm-2019-1111 31926073

[phy215204-bib-0059] VanHouten, J. N. , Dann, P. , Stewart, A. F. , Watson, C. J. , Pollak, M. , Karaplis, A. C. , & Wysolmerski, J. J. (2003). Mammary‐specific deletion of parathyroid hormone‐related protein preserves bone mass during lactation. Journal of Clinical Investigation, 112, 1429–1436. 10.1172/JCI200319504 PMC228471 14597768

[phy215204-bib-0060] VanHouten, J. N. , & Wysolmerski, J. J. (2003). Low estrogen and high parathyroid hormone‐related peptide levels contribute to accelerated bone resorption and bone loss in lactating mice. Endocrinology, 144, 5521–5529. 10.1210/en.2003-0892 14500568

[phy215204-bib-0061] Walther, D. J. , Peter, J. U. , Winter, S. , Holtje, M. , Paulmann, N. , Grohmann, M. , Vowinckel, J. , Alamo‐Bethencourt, V. , Wilhelm, C. S. , Ahnert‐Hilger, G. , & Bader, M. (2003). Serotonylation of small GTPases is a signal transduction pathway that triggers platelet alpha‐granule release. Cell, 115, 851–862. 14697203 10.1016/s0092-8674(03)01014-6

[phy215204-bib-0062] Weaver, S. R. , Fricke, H. P. , Xie, C. , Lipinski, R. J. , Vezina, C. M. , Charles, J. F. , & Hernandez, L. L. (2018). Peripartum fluoxetine reduces maternal trabecular bone after weaning and elevates mammary gland serotonin and PTHrP. Endocrinology, 159, 2850–2862. 10.1210/en.2018-00279 29893816 PMC6456925

[phy215204-bib-0063] Weaver, S. R. , Xie, C. , Charles, J. F. , & Hernandez, L. L. (2019). In utero and lactational exposure to the Selective Serotonin Reuptake Inhibitor fluoxetine compromises pup bones at weaning. Scientific Reports, 9, 238. 10.1038/s41598-018-36497-8 30659249 PMC6338725

[phy215204-bib-0064] Wysolmerski, J. J. (2010). Interactions between breast, bone, and brain regulate mineral and skeletal metabolism during lactation. Annals of the New York Academy of Sciences, 1192, 161–169. 10.1111/j.1749-6632.2009.05249.x 20392232 PMC3777748

[phy215204-bib-0065] Yadav, V. K. , Ryu, J. H. , Suda, N. , Tanaka, K. F. , Gingrich, J. A. , Schutz, G. , Glorieux, F. H. , Chiang, C. Y. , Zajac, J. D. , Insogna, K. L. , Mann, J. J. , Hen, R. , Ducy, P. , & Karsenty, G. (2008). Lrp5 controls bone formation by inhibiting serotonin synthesis in the duodenum. Cell, 135, 825–837. 10.1016/j.cell.2008.09.059 19041748 PMC2614332

[phy215204-bib-0066] Zhu, Y. , Goff, J. P. , Reinhardt, T. A. , & Horst, R. L. (1998). Pregnancy and lactation increase vitamin D‐dependent intestinal membrane calcium adenosine triphosphatase and calcium binding protein messenger ribonucleic acid expression. Endocrinology, 139, 3520–3524. 9681503 10.1210/endo.139.8.6141

