# Supplementary material for: Peripartal treatment with low‐dose sertraline accelerates mammary gland involution and has minimal effects on maternal and offspring bone
Source: Physiol Rep. 2022 Mar 2;10(5):e15204. doi: 10.14814/phy2.15204 (PMC8889862; doi:10.14814/phy2.15204)
Supplement: Supplementary file 1 — Table S1 [file PHY2-10-e15204-s001.docx]

| Supplemental Table 1. Primer Sequences for the studied genes quantified by real-time-PCR | | |
| --- | --- | --- |
| **Gene** | **Forward Primer (5’—3’)** | **Reverse** **Primer (5’—3’)** |
| *S15* | TTGAGAAAGGCCAAAAAGGA | GTTGAAGGTCTTGCCGTTGT |
| *Rsp9* | GGAGACCCTTCGAGAAGTCG | GGGGATCCTTCTCGTCTAGC |
| *K8* | ATCGAGATCACCACCTACCG | AAGCCAGGGCTAGTGAGTCC |
| *Tph1* | CCCGGAAATCAAAGCAAAG | CTTCCTTCGCAGTGAGCTG |
| *MaoA* | ACAGCAACACAGTGGAGTGG | GGAACATCCTTGGACTCAGG |
| *Sert* | ATCACGCTGGGTTTGGATAG | ATGACCACGATGAGCACAAA |
| *Pthlh* | TTCCTGCTCAGCTACTCCGT | GATGGACTTGCCCTTGTCAT |
| *Pthr1* | GGACAGATGGACCAAGAAGC | TTGAGCACAACACAGGAAGC |
| *Orai1* | ACCCCACGAGCGCATGCATC | GCTTGGTGGGGCTTGGCTGT |
| *Stim1* | TGCCTTCCTCTTACCTCTGC | ATGACTGTGGCTCCCATAGC |
| *Spca1* | AGGCAGAAGAAGCACCAAAA | TAACCAGCCAACCAACATGA |
| *Spca2* | CCTGTGCAACGAGAAACTGA | CCTGCAACGCCTTTATGATT |
| *Pmca1* | AACGACTGGAGCAAGGAGAA | CCGTACTTCACTTGGGCAAT |
| *Pmca2* | ACGTATGGGGACACTGAAGC | TTGCCCAAAAATCTGTTTCC |
| *Serca2* | TACTGACCCTGTCCCTGACC | CACCACCACTCCCATAGCTT |
| *5htr7* | ATCAACCGGAAGCTCTCTGC | CACAGTGGTCACAGTTTTGTAGC |
| *β-casein* | GGTGAATCTCATGGGACAGC | GAGATGGTTTGAGCCTGAGC |
| *α-lactalbumin* | CTGCCTCTGAGCCTTGTACC | GTAAAACCCCCATCGAGACC |
| *Cav1.3* | TCAGCGTCAGTGTGTGGAATA | CGAAAGGCGAGGAGTTCAC |
| *Trpv6* | TACAAGCCCAGCAGATTTCC | GCCTCCTACTTCACCTGTGG |
| *Calbindin D9K* | CCCGAAGAAATGAAGAGCATTTT | TTCTCCATCACCGTTCTTATCCA |
| *Ncx1* | CCCAGGACCAGTATGCAGAT | CATGGTAGATGGCAGCAATG |
| *Nfatc* | GAGTACACCTTCCAGCACCTT | TATGATGTCGGGGAAAGAGA |
| *Mmp9* | AAGGACGGTTGGTACAGGAA | GATCCTCAAAGGCGGAGTC |
| *Trap* | CGACAAGAGGTTCCAGGAGA | TGCCAAGGTGATCATGGTTT |
| *mCsf* | CGAATGTTCTCCCACTTCCT | TGGACAATCAAAGGCTGAGG |
| *Ctsk* | TGCTCCAGTCTGTCCATACG | CTATGGGAGTGGTGGGATGGs |
| *Rank* | CAGGACAGGGCTGATGAGAG | CCGCTAGAGATGAACGTGGA |
| *RankL* | GGAGGATGAAACAAGCCTTTG | ACATCCAACCATGAGCCTTC |
| *Opg* | AAGCTGGAACCCCAGAGC | GTGCTGCACTTCGTGTGTTT |
| *Runx2* | ATGCTTCATTCGCCTCACAAA | GCACTCACTGACTCGGTTGG |
| *Mmp13* | CCGAACTTAACTTACAGGATTG | GGTGTCACTCAGACCAGACC |
| *Alkp* | CTACGCACCCTGTTCTGAGG | GACCTCTCCCTTGAGTGTGG |
| *Bglap* | ACATGAGGGAGACAACAGGG | TTAGGAGACAGGGTGATGGC |
| *Bmp1* | AGCTAGTGAGTAGCCAGGGG | TGTTCACGAAAGCCTGACCG |
| Primers were designed using Primer 3. All primers were run at an annealing temperature of 60°C | | |

Abbreviations: S15, ribosomal protein S15; Rsp9, ribosomal protein S9; K8, cytokeratin 8; Tph1, tryptophan hydroxylase 1; MaoA, monoamine oxidase A; Sert, serotonin reuptake transporter; Pthlh, parathyroid hormone related protein; Orai1, calcium release-activated calcium channel protein 1; Stim1, stromal interaction molecule 1; Spca1 and 2, calcium-transporting ATPase type 2C member 1 and 2; Pmca1 and 2, plasma membrane calcium ATPase 1 and 2; Serca2, sarco/endoplasmic reticulum calcium ATPase 2; 5htr7, serotonin receptor 7; Cav1.3, calcium channel, voltage-dependent, L-type, alpha 1D subunit; Trpv6, transient receptor potential cation channel subfamily V member 6; Ncx1, sodium/calcium exchanger 1; Nfatc, nuclear factor of activated T-cells, cytoplasmic 1; Mmp9 and 13, matrix metallopeptidase 9 and 13; Trap, tartrate-resistant acid phosphatase; mCsf, macrophage colony stimulating factor 1; Ctsk, cathepsin K; Rank, receptor activator of nuclear factor kappa-B; RankL, receptor activator of nuclear factor kappa-B ligand; Opg, osteoprotegerin; Runx2, runt-related transcription factor 2; Alkp, alkaline phosphatase; Bglap, bone gamma-carboxyglutamate protein; Bmp1, bone morphogenic protein 1
