# Supplementary figures and images for: Peripartal treatment with low‐dose sertraline accelerates mammary gland involution and has minimal effects on maternal and offspring bone
Source: Physiol Rep. 2022 Mar 2;10(5):e15204. doi: 10.14814/phy2.15204 (PMC8889862; doi:10.14814/phy2.15204)

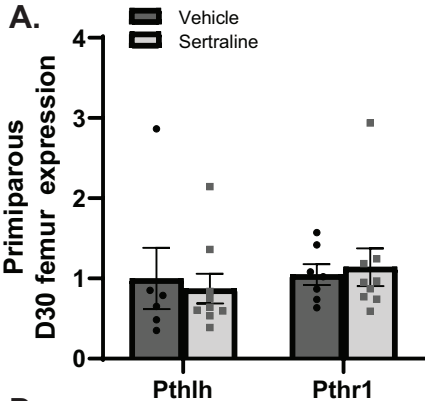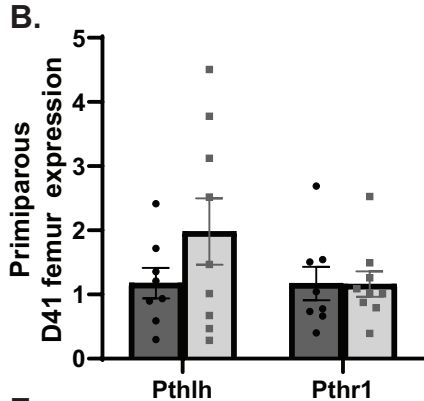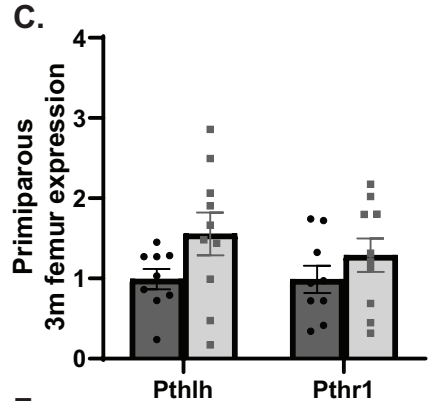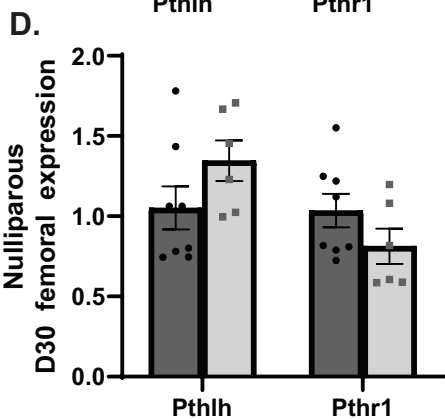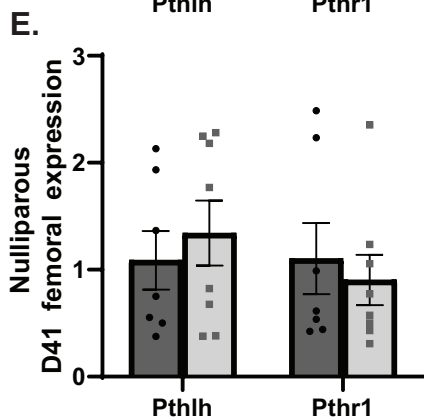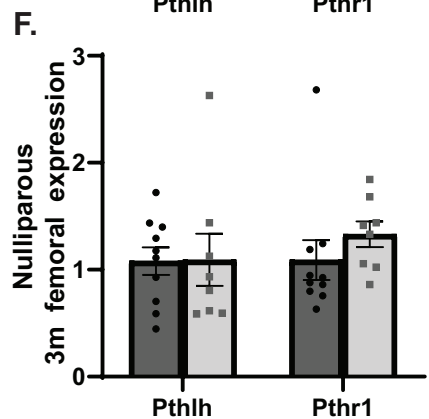

Supplement: Supplementary file 2 — Figure S1 [file PHY2-10-e15204-s002.pdf]

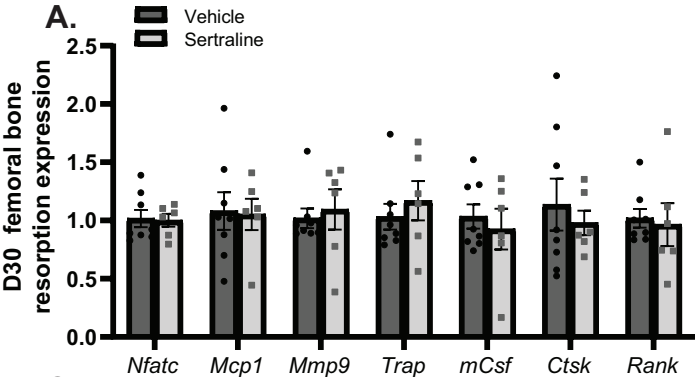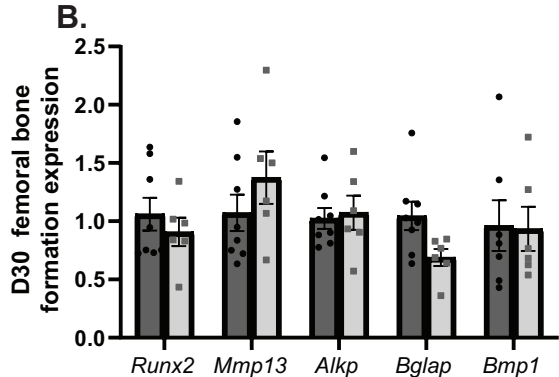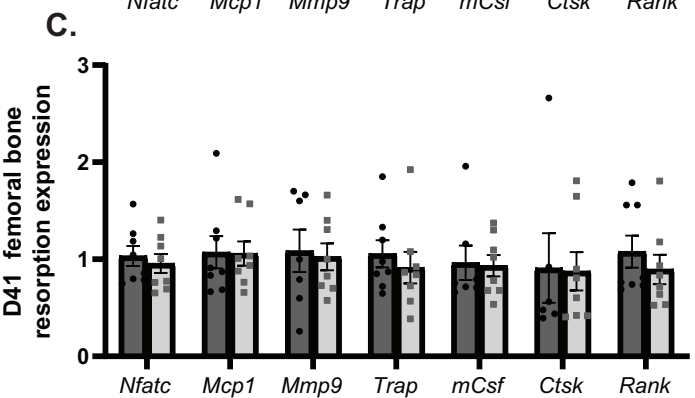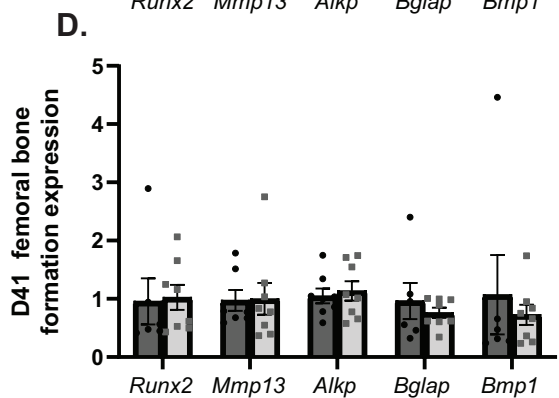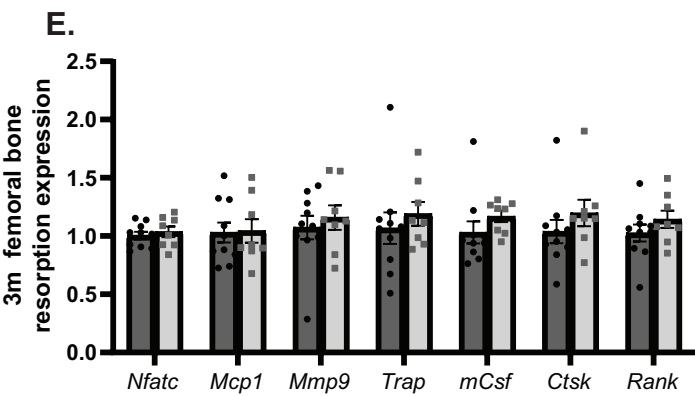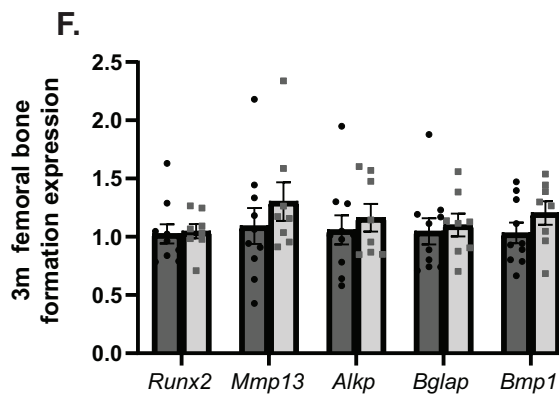

Supplement: Supplementary file 3 — Figure S2 [file PHY2-10-e15204-s003.pdf]
